# Water Vapor‐Enhanced Selective Production of Methane During Photothermal CO_2_ Reduction: Mechanistic Insights Into Boron‐Doped Nickel Catalysts

**DOI:** 10.1002/advs.202519611

**Published:** 2025-11-16

**Authors:** Cong Wan, Yiming Li, Min Liu, Honglei Zhang, Jianwen Zhang, Chengrui Xie, Huiwen Zhu, Zijun Yan, Chengheng Pang, Tao Wu

**Affiliations:** ^1^ Department of Chemical and Environmental Engineering University of Nottingham Ningbo 315100 China; ^2^ Municipal Key Laboratory of Clean Energy Technologies of Ningbo University of Nottingham Ningbo China Ningbo 315100 China

**Keywords:** boron‐doped Ni, carbon dioxide reduction, CH_4_ selectivity, hydrogen, photothermal catalysis, water vapor

## Abstract

The development of efficient CO_2_ reduction technologies is crucial for mitigating climate change and advancing sustainable energy systems. In this study, the photothermal catalytic reduction of CO_2_ to methane (CH_4_) using boron‐doped Ni as a catalyst, focuses on enhancing product selectivity through reaction parameter optimization. Notably, addition of water vapor substantially improves both CH_4_ yield (195.85 mmol g·h^−1^) and selectivity (80.21%), representing a significant advancement over traditional approaches. Through integrated experimental characterization and density functional theory (DFT) calculations, the underlying mechanism involving competitive adsorption dynamics is elucidated between CO_2_ and H_2_O molecules on the boron‐doped Ni surface, along with their parallel dissociation pathways. DFT calculations also confirm that boron doping upshifts the Ni d‐band center, strengthening CO adsorption for subsequent hydrogenation. In situ diffuse reflectance infrared Fourier transform spectroscopy (DRIFTS) measurements indicated that the reverse water‐gas shift (RWGS) reaction on boron‐doped Ni proceeded primarily via a dissociation pathway. Furthermore, the introduction of H_2_O enables alternative CO_2_ reduction mechanisms through the carboxylate and bicarbonate pathways. This study demonstrates the significant potential of boron‐doped Ni catalysts for enhanced CO_2_ methanation and provides valuable mechanistic insights into how reaction parameters influence the photothermal CO_2_ reduction process.

## Graphical abstract

Boron‐doped nickel catalysts achieve exceptional photothermal CO_2_ methanation performance, reaching 195.85 mmol g^−1^ h^−1^ CH_4_ yield with 80.21% selectivity through optimized H_2_/H_2_O feeding. Boron doping modulates the Ni d‐band center, strengthening CO adsorption and facilitating subsequent hydrogenation. This work provides atomic‐level insights into photothermal catalytic mechanisms and pathway control for sustainable CO_2_ conversion.

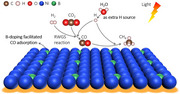

## 1 Introduction

Rising atmospheric CO_2_ concentration and the global climate response are inextricably linked, leading to regional temperature extremes and increased precipitation events.^[^ ^1^ ^]^ Achieving a net‐zero carbon society has become critical for mitigating these impacts. In this context, the capture and utilization of CO_2_ for synthesizing valuable chemicals—including hydrocarbon fuels, commodity chemicals, and building materials—has attracted considerable research attention worldwide.^[^ ^2^, ^3^, ^4^ ^]^

Among various CO_2_ utilization strategies, CO_2_ methanation offers a promising pathway for transforming CO_2_ into hydrocarbon fuels, providing a dual solution to carbon emission reduction and CO_2_ storage/transportation challenges.^[^ ^4^, ^5^ ^]^ This reaction typically requires temperatures around 300 °C, which can be efficiently achieved through concentrated solar irradiation. The photothermal effect not only provides the necessary thermal energy but also enhances reactant molecular kinetics and promotes CO_2_ activation,^[^ ^6^ ^]^ making solar energy an ideal sustainable energy source for CO_2_ hydrogenation.^[^ ^7^, ^8^ ^]^ Consequently, photothermal CO_2_ methanation has emerged as a particularly attractive approach for renewable fuel production, which also serves as an important carbon mitigation technology.^[^ ^9^, ^10^ ^]^

The success of photothermal CO_2_ methanation largely depends on catalyst selection, with Group VIII metals (Ru, Rh, Ni, Co) exhibiting exceptional photothermal performance for CO_2_ conversion. These metals demonstrate efficient solar energy utilization across the spectrum due to localized surface plasmon resonance (LSPR) effects.^[^ ^11^, ^12^, ^13^, ^14^ ^]^ Theoretical calculations further confirm their strong capability for CO_2_ chemisorption and dissociation into CO and *O.^[^ ^15^, ^16^ ^]^ Among these metals, Ni‐based catalysts emerge as particularly promising candidates for CO_2_ methanation, offering an optimal balance of catalytic activity, stability, and cost‐effectiveness.^[^ ^17^, ^18^, ^19^ ^]^ Recent advances have explored various strategies to enhance the photothermal CO_2_ reduction performance of Ni catalysts, including the design of core‐shell structures (CO selectivity > 80%),^[^ ^20^, ^21^ ^]^ single‐atom configurations (CH_4_ yield = 1.043 mmol g·h^−1^),^[^ ^22^ ^]^ metal clusters (CO selectivity = 100%),^[^ ^23^ ^]^ and precious metal doping (CH_4_ yield = 6.76 mmol g·h^−1^).^[^ ^24^, ^25^ ^]^ However, current Ni‐based catalysts still face challenges in achieving satisfactory CH_4_ yield and selectivity through photothermal catalytic reduction. Therefore, developing high‐performance yet economical Ni‐based catalysts for efficient CO_2_‐to‐CH_4_ conversion remains a crucial research direction.

One promising approach to enhance Ni catalyst performance is through strategic elemental doping. In particular, boron doping effectively modulates catalytic activity in diverse systems by altering electronic structure, charge transport, and phase behavior, frequently enhancing activity and selectivity in transition metal catalysts.^[^ ^26^, ^27^, ^28^ ^]^ In the specific case of Ni catalysts, boron doping induces a pronounced charge transfer from boron to nickel atoms. This electronic redistribution manifests as a downshift of the Ni d‐band center, elevating the Fermi level and consequently optimizing the binding energies of key reaction intermediates.^[^ ^29^, ^30^ ^]^ Such electronic tuning proves particularly advantageous for CO_2_ hydrogenation processes, where precise control of intermediate adsorption strengths is crucial for selective methane formation.

Beyond catalyst design, the choice of reducing agents also critically influences the efficiency of CO_2_ conversion processes. Hydrogen (H_2_) and water (H_2_O) are among the most commonly used reducing agents in photothermal CO_2_ conversion, each offering distinct advantages and limitations. While H_2_O offers practical advantages as a more economical and convenient reductant, thermodynamic constraints limit its effectiveness: the reduction potential for H_2_ evolution from H_2_O (0 V versus SHE) is more favorable than that for CO_2_ reduction (−1.9 V versus SHE), leading to preferential H_2_O splitting over CO_2_ activation under photochemical conditions.^[^ ^31^ ^]^ Notably, the reaction kinetics reveal a striking disparity: CO_2_ hydrogenation with H_2_ proceeds approximately three orders of magnitude faster than with H_2_O,^[^ ^8^ ^]^ demonstrating H_2_ as the dominant active species in CO_2_ reduction in the presence of H_2_O.

The mechanistic interplay between H_2_, H_2_O, and CO_2_ on catalyst surfaces further complicates the reaction landscape. During CO_2_ dissociation on Ni, *H_2_O and *CO species generated via the RWGS reaction establish a quasi‐equilibrium with gaseous CO.^[^ ^32^, ^33^ ^]^ These adsorbed *CO and *H_2_O layers critically influence the availability of contiguous bare metal sites, which are necessary for CO_2_ and CO activation. Consequently, it also affect CH_4_ yield and selectivity on Ni, regardless of whether CO or H_2_O are introduced as reactants or produced in situ.^[^ ^34^, ^35^, ^36^ ^]^ However, the mechanisms underlying this phenomenon and strategies to enhance CO_2_ reduction selectivity warrant further investigation, particularly regarding the combined effects of H_2_O and H_2_ as reducing agents on catalyst activity and product distribution, underscoring the need for systematic investigation of their combined effects on product distribution in photothermal CO_2_ methanation.

Integrating these considerations of catalyst design and reaction environment, this study aims to enhance CH_4_ selectivity in photothermal catalytic CO_2_ reduction by systematically investigating reaction performance under various conditions using boron‐doped Ni catalysts. We specifically examine the synergistic effects between thermal and photothermal energy on catalytic performance, focusing on their influence on product distribution. Additionally, we investigate how hydrogen and water vapor concentrations affect CO_2_ reduction efficiency and CH_4_ selectivity. Through complementary experimental techniques and theoretical calculations, we elucidate the underlying mechanisms governing the enhanced catalytic performance, contributing to the development of more effective strategies for photothermal CO_2_ methanation.

## 2 Results and Discussion

### 2.1 Characterization of the Catalysts

**Figure 1:**
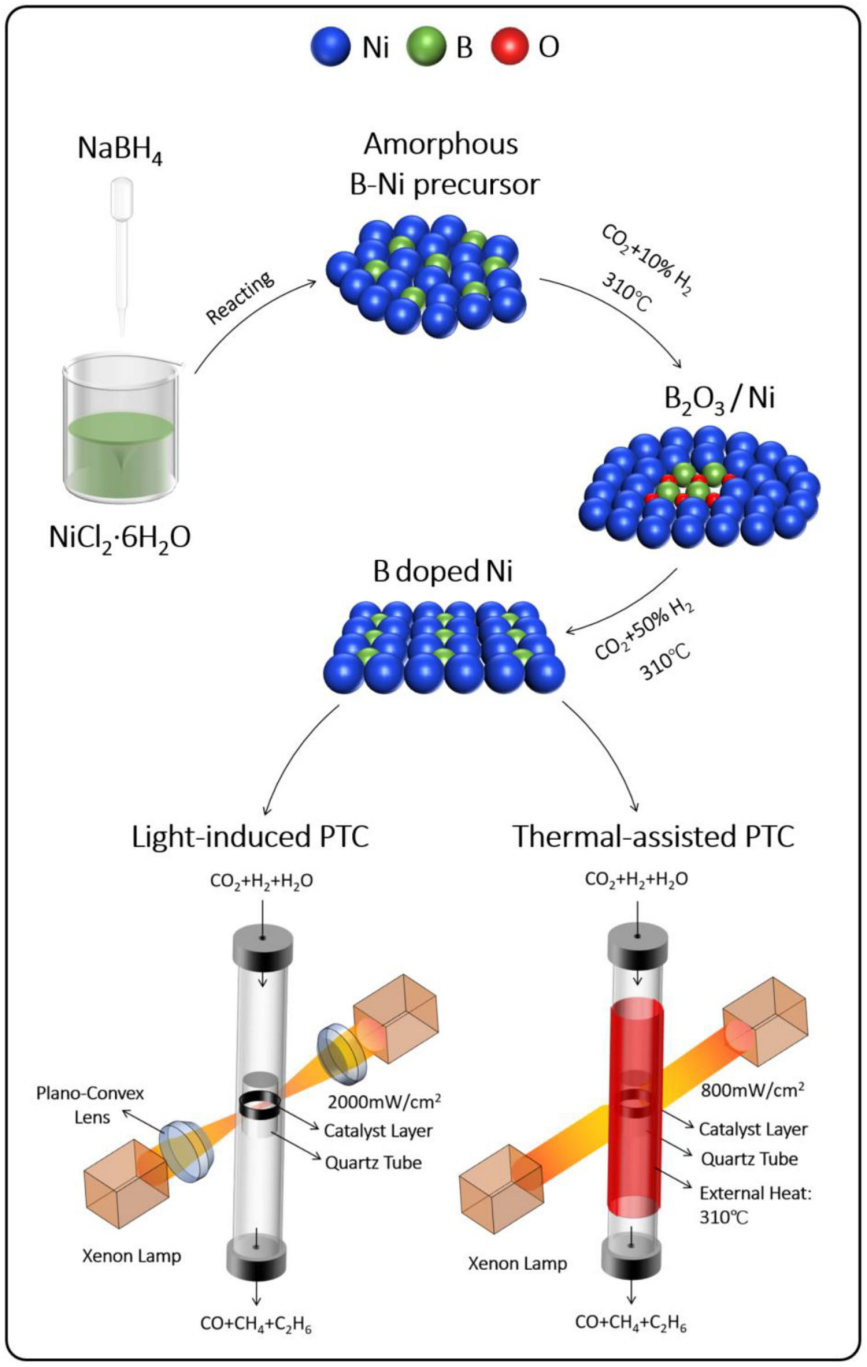
Schematic diagram of the catalyst preparation and performance testing.

**Figure 2:**
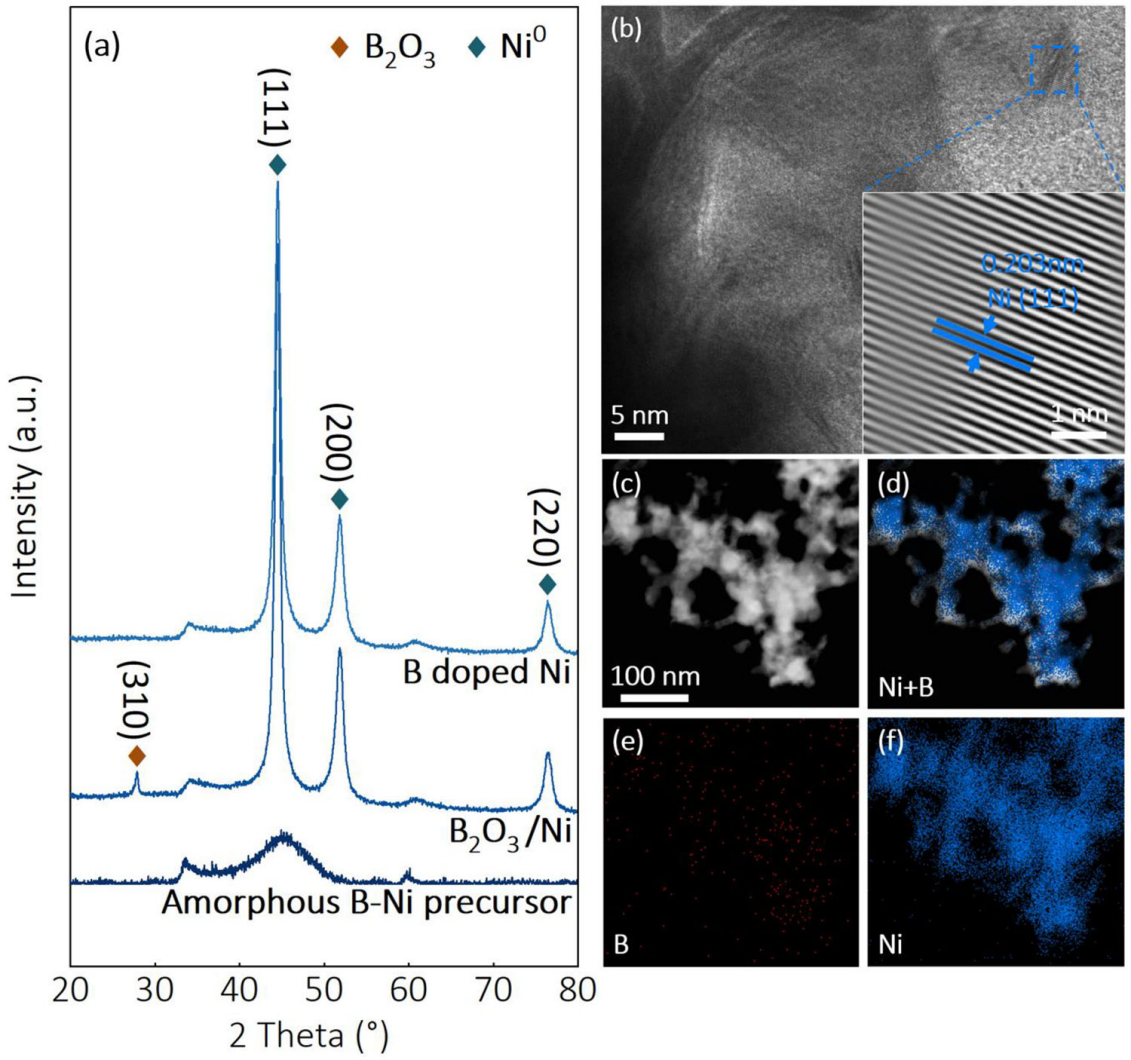
a) XRD pattern of the Amorphous B‐Ni precursor, B_2_O_3_/Ni, and B doped Ni; b) HRTEM image, and c–e) HAADF image and the element mapping of B and Ni of B doped Ni.

The boron‐doped Ni catalyst was synthesized through a reduction reaction between NaBH_4_ and NiCl_2_, followed by thermal treatment in a CO_2_‐H_2_ atmosphere (**Figure** **1**, see details in Experimental Section). X‐ray diffraction (XRD) analysis revealed the crystalline phase evolution of the materials (**Figure** **2**a). The precursor exhibited weak diffraction peaks corresponding to both metallic Ni and B_2_O_3_ phases. After annealing in CO_2_ with 10% H_2_, the XRD pattern showed well‐defined Ni diffraction peaks coexisting with crystalline B_2_O_3_ phases, indicating thermally induced crystallization and phase segregation that created abundant B_2_O_3_/Ni interfaces. Notably, treatment under CO_2_ with 50% H_2_ yielded XRD patterns containing only Ni reflections, with the (111) plane as the dominant crystallographic orientation, suggesting complete reduction of B_2_O_3_ to boron species incorporated into the Ni lattice.

The Scanning electron microscopy (SEM) images revealed that the B_2_O_3_/Ni sample consisted of aggregated nanoflakes ranging from tens to hundreds of nanometers in size (Figure S1a, Supporting Information). Transmission electron microscopy (TEM) showed that both the amorphous precursor and the annealed samples exhibited similar morphologies (Figure S2, Supporting Information). High‐resolution TEM (HRTEM) images of B_2_O_3_/Ni (Figure S1b, Supporting Information) confirmed the presence of interfaces between two distinct crystalline phases: one with a lattice spacing of 0.32 nm corresponding to the (310) plane of B_2_O_3_ (Figure S1c, Supporting Information), and another with a spacing of 0.203 nm corresponding to the (111) plane of face‐centered cubic Ni (Figure S1d, Supporting Information). Scanning transmission electron microscopy with energy dispersive X‐ray spectroscopy (STEM‐EDX) revealed homogeneous distribution of Ni and B elements throughout the sample (Figure S1f, Supporting Information).

High‐resolution transmission electron microscopy (HRTEM) analysis of the boron‐doped Ni catalyst (Figure 2b) revealed exclusively the (111) crystallographic plane of metallic nickel, providing direct evidence for the complete reduction of B_2_O_3_ to boron during the 50% H_2_ treatment process. Complementary energy‐dispersive X‐ray spectroscopy (EDS) elemental mapping (Figure 2d) confirmed the homogeneous distribution of both nickel and boron throughout the sample, demonstrating successful and uniform boron incorporation into the nickel matrix.

X‐ray photoelectron spectroscopy (XPS) characterization was conducted to determine the elemental composition and chemical states of the catalytic materials. High‐resolution Ni 2p spectra (Figure S3a,b, Supporting Information) revealed three characteristic peaks for all samples (B‐Ni precursor, B_2_O_3_/Ni, and B‐doped Ni): a metallic Ni peak at 852.5 eV, along with Ni^2^⁺ signatures at 855.57 eV (Ni 2p_3_/_2_) and 873.25 eV (Ni 2p_1_/_2_), indicative of surface oxidation upon air exposure. The B 1s spectra confirmed successful boron incorporation into the nickel matrix. Notably, the absence of sodium and chlorine signals in both pre‐ and post‐reaction samples demonstrates the selective incorporation of boron during synthesis, with complete removal of other precursor elements.^[^ ^37^, ^38^ ^]^

X‐ray absorption spectroscopy (XAS) characterization of the boron‐doped Ni catalyst reveals key structural insights through complementary XANES and EXAFS analyses.^[^ ^39^ ^]^ The XANES spectrum (Figure S3c, Supporting Information) demonstrates that B‐doped Ni maintains predominantly metallic character, shown by its absorption edge energy matching the Ni foil reference (Ni⁰), with only minor pre‐edge features indicating trace surface oxidation. EXAFS analysis (Figure S3d, Supporting Information) confirms preservation of the metallic Ni framework through the dominant Ni‐Ni coordination peak at an identical bond distance to the Ni foil reference, demonstrating that boron incorporation occurs without significant lattice distortion. These results show that the catalyst largely maintains its bulk metallic nickel properties with surface Ni oxidized, whereas boron doping primarily affects the local electronic environment rather than disrupting the overall lattice structure.

### 2.2 Thermal and Photothermal Reduction of CO_2_ with H_2_

**Figure 3:**
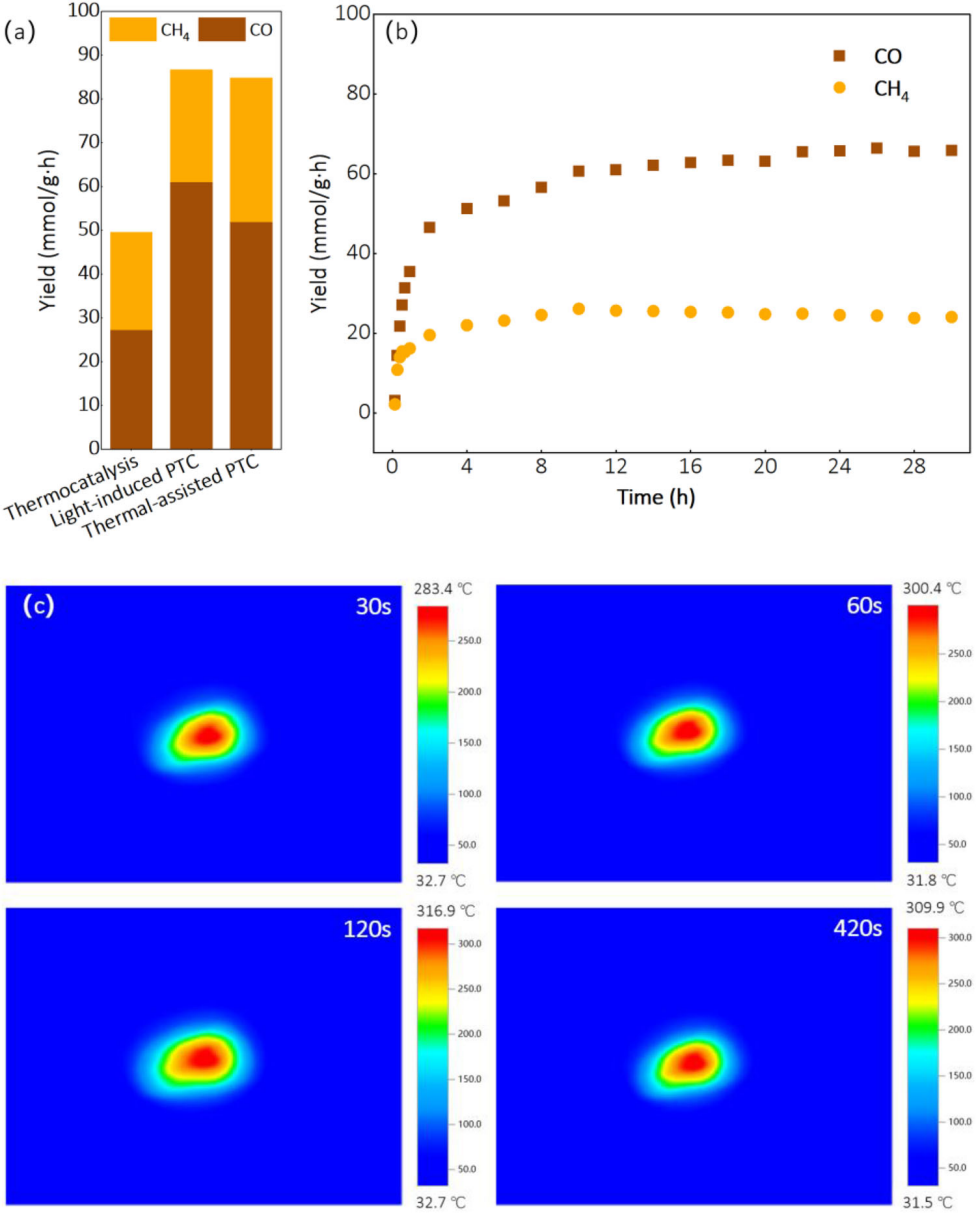
a) CO and CH_4_ yield at dark, light‐induced photothermal catalytic reduction(2000 mW cm^−2^) and thermal‐assisted photothermal catalytic reduction (800 mW cm^−2^) reaction with 10% H_2_ at steady state, b) catalytic performance of B doped Ni in a 30 h continuous test under focused full‐spectrum light irradiation (2000 mW cm^−2^), c) B doped Ni surface temperature under focused light illumination from Xe arc lamp at 2000 mW cm^−2^_._

Light‐induced photothermal catalytic (PTC) and thermally assisted photothermal catalytic reduction experiments were conducted to evaluate catalyst performance. **Figure** **3**c illustrates the temperature profile of boron‐doped Ni under focused light irradiation. The catalyst temperature rapidly reached 283.4 °C within 30 seconds and stabilized between 309.9 °C and 316.9 °C from 60 to 420 seconds. Accordingly, the external heating temperature for thermally assisted experiments was set to 310 °C.

Figure 3a displays the CO and CH_4_ production yields from CO_2_ reduction with 10% H_2_ under various reaction conditions. Under dark conditions (thermal catalysis alone), the system yielded 27.26 mmol g·h^−1^ of CO and 22.35 mmol g·h^−1^ of CH_4_ at steady state, corresponding to a CH_4_ selectivity of 45.05%. When light‐induced PTC was applied, we observed a notable increase in CO yield to 61 mmol g·h^−1^, while CH_4_ production increased slightly to 25.65 mmol g·h^−1^, reducing CH_4_ selectivity to 29.6%.

Thermally assisted PTC conditions demonstrated enhanced performance, achieving a CH_4_ yield of 32.88 mmol g·h^−1^. This improvement can be attributed to the more uniform temperature distribution in the reactor when external heating is applied, compared to the localized heating (≈310 °C only at the illumination center) in light‐induced PTC. The boosted yields of both CO and CH_4_ under light‐assisted conditions suggest a synergistic mechanism combining thermal catalysis (from photo‐induced heating) and photocatalysis (from generated charge carriers).^[^ ^40^, ^41^ ^]^

To evaluate the catalyst's stability, we performed a 30‐hour continuous reaction under light‐induced photothermal conditions (Figure 3b). The catalytic activity exhibited a gradual increase during the initial 10 hours, likely due to improved heat distribution from the illuminated center to the reactor periphery. Subsequently, the system maintained a stable CO production rate throughout the remaining duration of the experiment, confirming the outstanding long‐term stability of the boron‐doped Ni catalyst.

### 2.3 H_2_ Enhanced CH_4_ Yield and Selectivity

**Figure 4:**
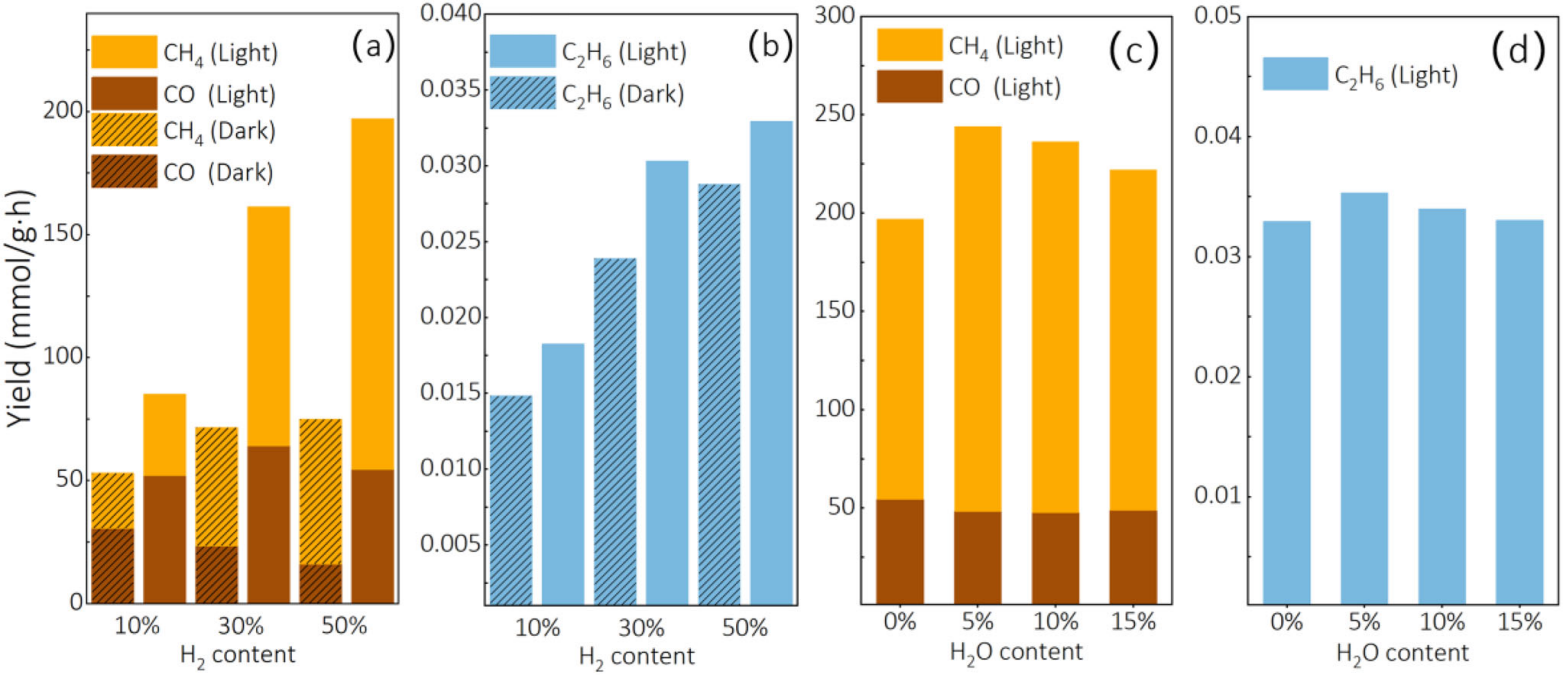
a)CO, CH_4_, b)C_2_H_6_ yield at dark, thermal‐assisted photothermal catalytic reduction (800 mW cm^−2^) reaction on B doped Ni with different H_2_ content, c) CO, CH_4_, d) C_2_H_6_ yield by thermal‐assisted photothermal catalytic reduction (800 mW cm^−2^) reaction on B doped Ni with different H_2_O content.

**Figure 5:**
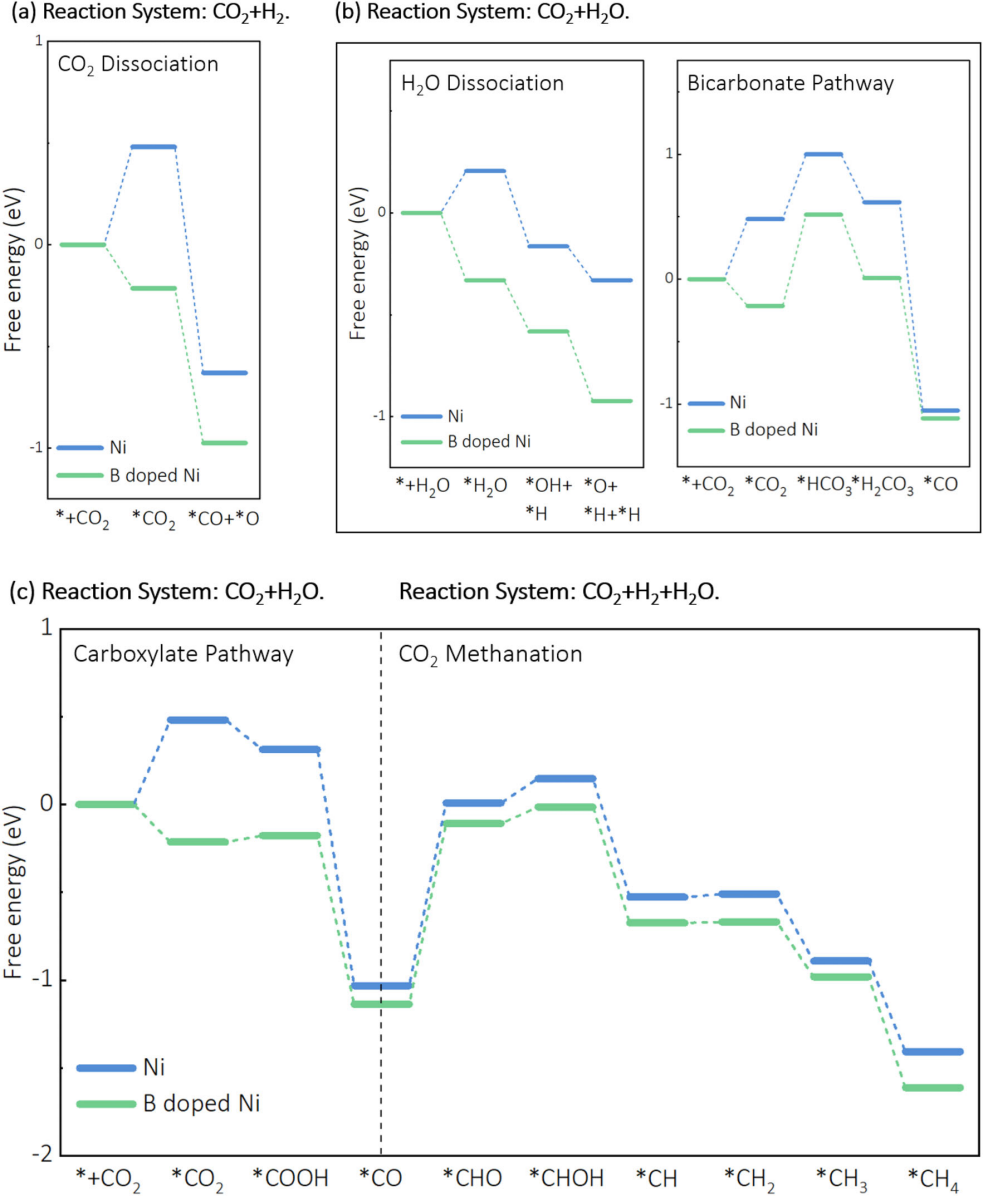
DFT calculated potential energy profiles for a) CO_2_ dissociation, b) H_2_O dissociation and CO_2_ reduction through bicarbonate intermediates, c) CO_2_ reduction through carboxylate intermediates and CO_2_ methanation over Ni(111), and B doped Ni(111) surface.

**Figure 6:**
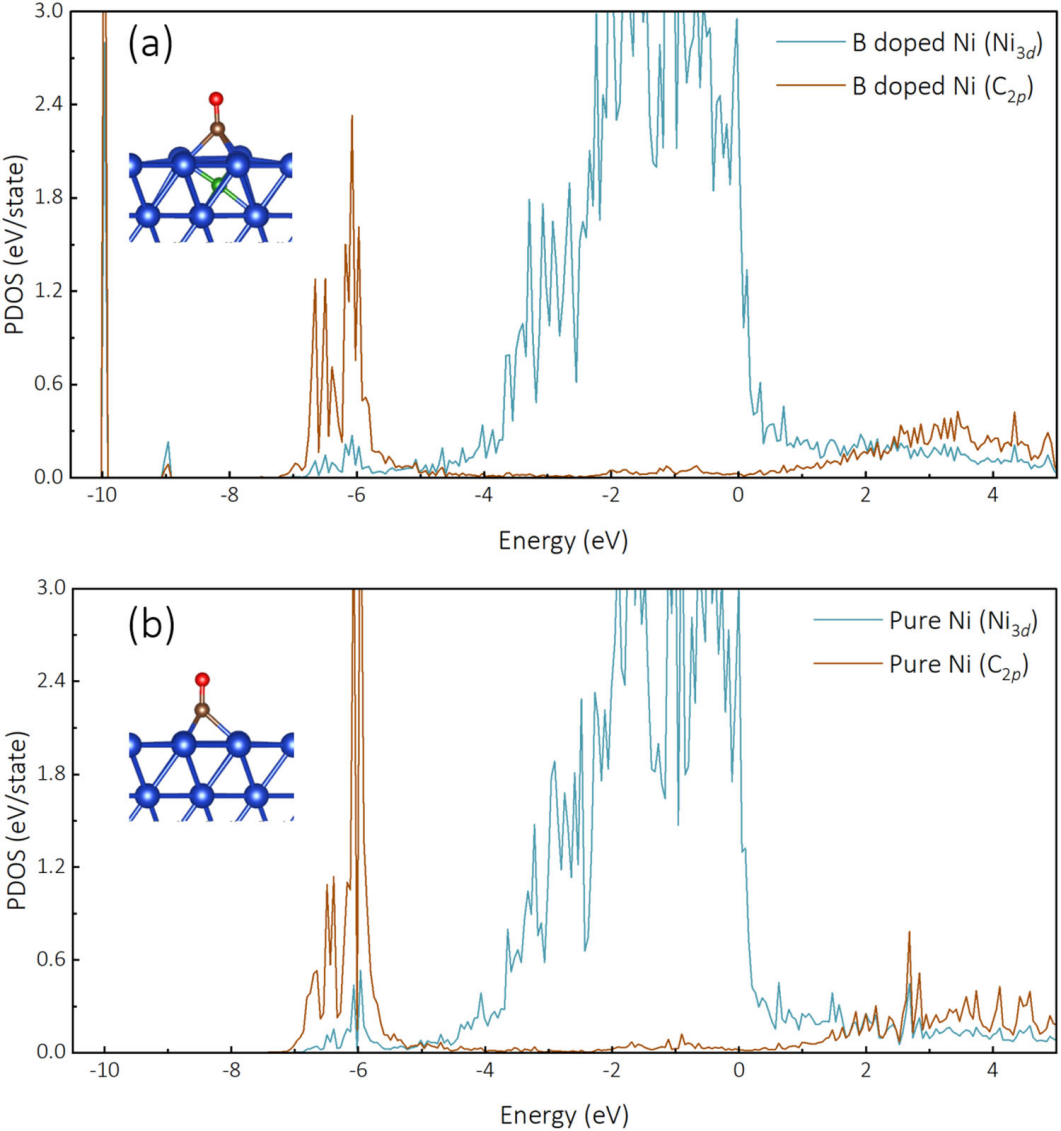
PDOS plot of Ni_3_ *_d_* and C_2_ *_p_* orbitals in a) pure Ni and b) B doped Ni catalysts with CO adsorption.

To further enhance CH_4_ productivity and selectivity, experiments were conducted with varying H_2_ concentrations and the introduction of H_2_O (**Figure** **4**a,b). For CO_2_ reduction with 10%, 30%, and 50% H_2_ without light illumination, the CO yield decreased slightly as H_2_ content increased (27.26, 23.39, and 16.03 mmol g·h^−1^, respectively), indicating that higher H_2_ concentrations facilitate the consumption of CO intermediates through subsequent hydrogenation steps. This mechanistic shift is directly supported by our DFT calculations (**Figure** **5**d), which show more favorable energetics for CO hydrogenation on B‐doped Ni surfaces. In contrast, the CH_4_ yield increased substantially with higher H_2_ concentrations, reaching 22.35, 48.12, and 58.87 mmol g·h^−1^, respectively, corresponding to CH_4_ selectivities of 45.05%, 67.3%, and 78.6%. This trend aligns with our computational findings that B‐doping enhances CO adsorption strength through greater orbital overlap between Ni 3d and C 2p states (**Figure** **6**), creating an optimal surface environment for CO hydrogenation when sufficient H_2_ is available. Under light illumination, CH_4_ productivity was further enhanced to 33.19, 97.15, and 142.57 mmol g·h^−1^ for the respective H_2_ concentrations, with CH_4_ selectivities of 38.99%, 60.25%, and 72.34%. The photo‐enhancement effect becomes more pronounced at higher H_2_ concentrations (2.4‐fold increase at 50% H_2_ versus 1.5‐fold at 10% H_2_), suggesting that light‐generated charge carriers and localized heating synergistically activate both CO_2_ and H_2_ molecules on the catalyst surface, accelerating the rate‐determining hydrogenation steps in the methanation pathway.

### 2.4 Steam Enhanced Photothermal Reduction of CO_2_ with H_2_

**Figure 7:**
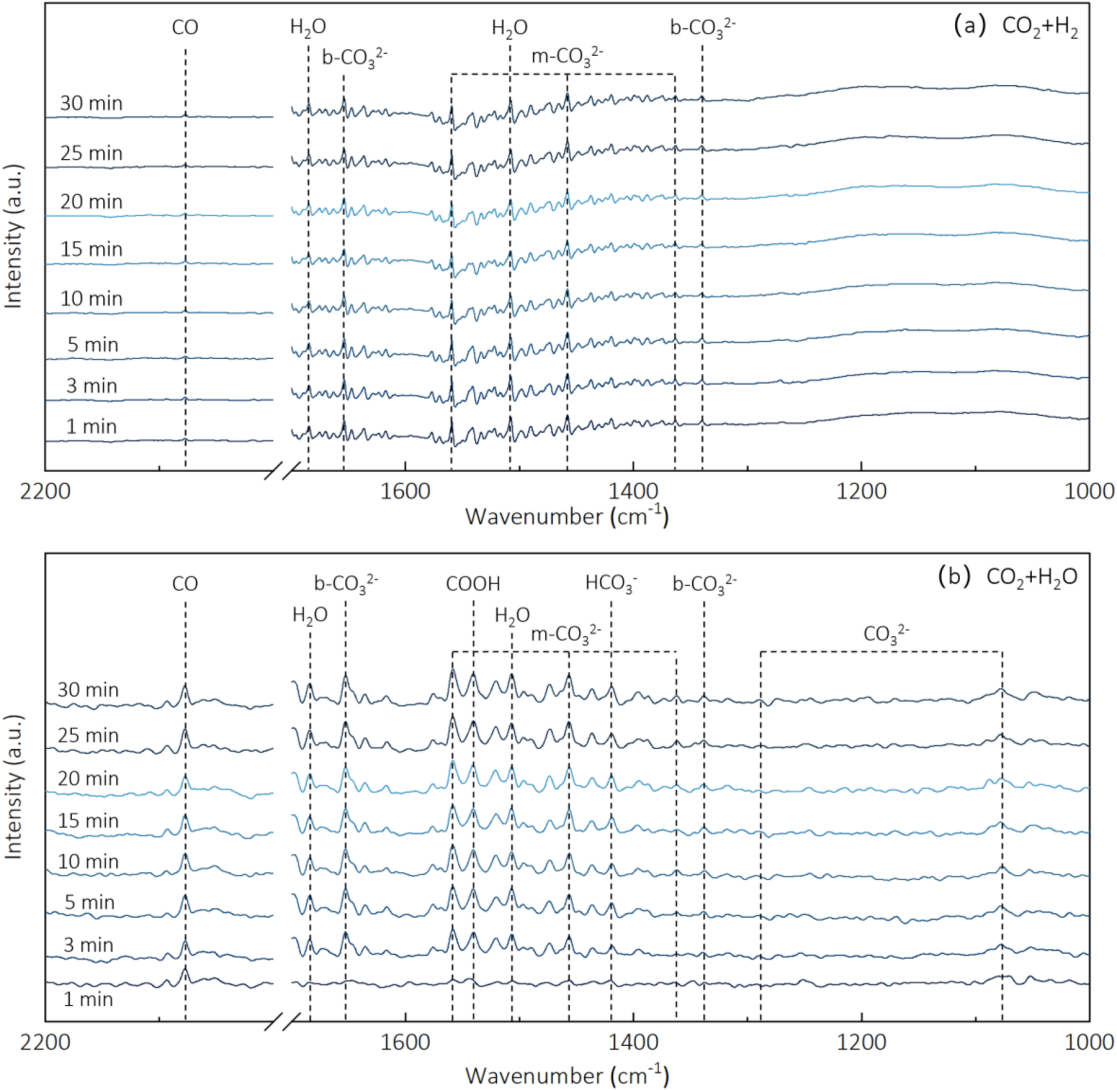
In situ DRIFTS spectra of a) CO_2_ reduction with H_2_, b) CO_2_ reduction with H_2_O on B doped Ni.

The introduction of H_2_O into the CO_2_/H_2_ reaction system provides an additional hydrogen source through dissociation on the catalyst surface, as confirmed by our DFT calculations showing favorable energetics for H_2_O dissociation on B‐doped Ni (Figure 5b). This additional hydrogen supply facilitates the reduction of CO to CH_4_ and enhances CH_4_ selectivity through a dual‐pathway mechanism. Compared to CO_2_ reduction with 50% H_2_ alone, the photothermal catalytic reduction of CO_2_ with 50% H_2_ and 5% H_2_O achieved significantly higher CH_4_ yield and selectivity (Figure 4c,d: 195.85 mmol g·h^−1^ and 80.21%, respectively). This 37% increase in CH_4_ yield (from 142.57 to 195.85 mmol g·h^−1^) directly correlates with our in situ DRIFTS observations showing enhanced formation of reaction intermediates (*COOH at 1541 cm^−1^ and HCO_3_^−^ at 1410 cm^−1^) when H_2_O is present (**Figure** **7**b). Meanwhile, the CO yield decreased slightly to 48.33 mmol g·h^−1^ despite the activation of additional CO_2_ reduction pathways, indicating more efficient conversion of CO intermediates to CH_4_ through the hydrogen‐rich environment created by H_2_O dissociation. The observed enhancement in both activity and selectivity demonstrates a clear synergistic effect between H_2_ and H_2_O, where H_2_ provides the primary reducing power while H_2_O enables alternative CO_2_ activation routes and supplies additional surface hydrogen species. Compared with other advanced photothermal catalysts published in the literature, the B‐doped Ni catalyst has an excellent performance in terms of the CH_4_ yield and selectivity (**Table** **1**).

However, higher water vapor content (>5%) was found to inhibit CO_2_ reduction, as evidenced by the 32% decrease in CH_4_ yield when H_2_O concentration increased from 5% to 10% (Figure S4, Supporting Information). This inhibition effect can be mechanistically explained by competitive adsorption between CO_2_ and H_2_O on the Ni surface, which is quantitatively supported by our DFT calculations showing comparable adsorption energies for both molecules on B‐doped Ni sites (Figure 5a,b). At higher H_2_O concentrations, water molecules preferentially occupy active sites on the catalyst surface, blocking CO_2_ access and impeding the methanation pathway. Furthermore, the boron‐doped Ni catalyst dissociates both CO_2_ and H_2_O, generating oxygen atoms that react with zero‐valent Ni to form NiO, as confirmed by post‐reaction XPS analysis showing increased Ni^2^⁺ signals at 855.57 eV (Figure S3a, Supporting Information). Since NiO exhibits limited photocatalytic performance due to its wider band gap and poor charge carrier mobility, light illumination had minimal effect on the reduction of CO_2_ and H_2_O mixtures at higher H_2_O concentrations (Figure S4, Supporting Information), further confirming the mechanistic shift from a synergistic to a competitive regime at excessive H_2_O levels.

**Table 1:** Comparison of photothermal catalysts for CO_2_ reduction used in this work with other catalysts reported in literature.

Catalyst	Reactor Parameter	Gas composition	Yield[mmol g_cat_·h^−1^]	Reference
B doped Ni	Flow, external heat, 310 °C, 800 mW cm^−2^	H_2_:CO_2_=1:1, H_2_O: 5%, 10 ml min^−1^	CH_4_=195.85, CO=48.33, C_2_H_6_=0.035	This Work
Ni/C‐In_2_O_3_	Flow, 352 °C1521.9 mW cm^−2^	H_2_:CO_2_:N_2_=4:1:5, 20 ml min^−1^	CO=20.96	[23]
CuCo_y_BO_x_	Flow, 300 °C, external heat, 500 mW cm^−2^	H_2_:CO_2_=4:1, 50 ml min^−1^	CO=124.7	[42]
In_2_O_3_@Ni	Batch, 250 °C, 257.4 mW cm^−2^	H_2_:CO_2_=4:1	CH_4_=1.043	[22]
Ni–Ru/HZSM‐5	Flow, 3500 mW cm^−2^	H_2_:CO_2_=4:1, 5 ml min^−1^	CH_4_=6.76, CO=0.8	[24]
Ru‐TiO_x_	Batch, 1000 mW cm^−2^	H_2_:CO_2_=4:1	CH_4_=15.84	[43]
Ir‐CoO/Al_2_O_3_	Flow, 2000 mW cm^−2^	H_2_:CO_2_=4:1, 20 ml/min	CH_4_=128.9	[44]
MnNiZrRuCe HEMG	Flow, 300 °C, 1140 mW cm^−2^	H_2_:CO_2_=1:1, 5 ml min^−1^	CH_4_≈51	[45]
In_2_O_3‐x_/In_2_O_3_	Flow, 2000 mW cm^−2^	H_2_:CO_2_=1:1, 1 ml min^−1^	CO≈1.9	[46]
S_Fe‐Ni_	Batch,3330 mW cm^−2^	H_2_:CO_2_=1:1	CO=44.1	[21]
c‐TiO_2_@a‐TiO_2‐x_(OH)_y_	Batch, 4000 mW cm^−2^	H_2_:CO_2_=1:1	CO≈5.1	[47]

### 2.5 DFT Calculations

**Figure 8:**
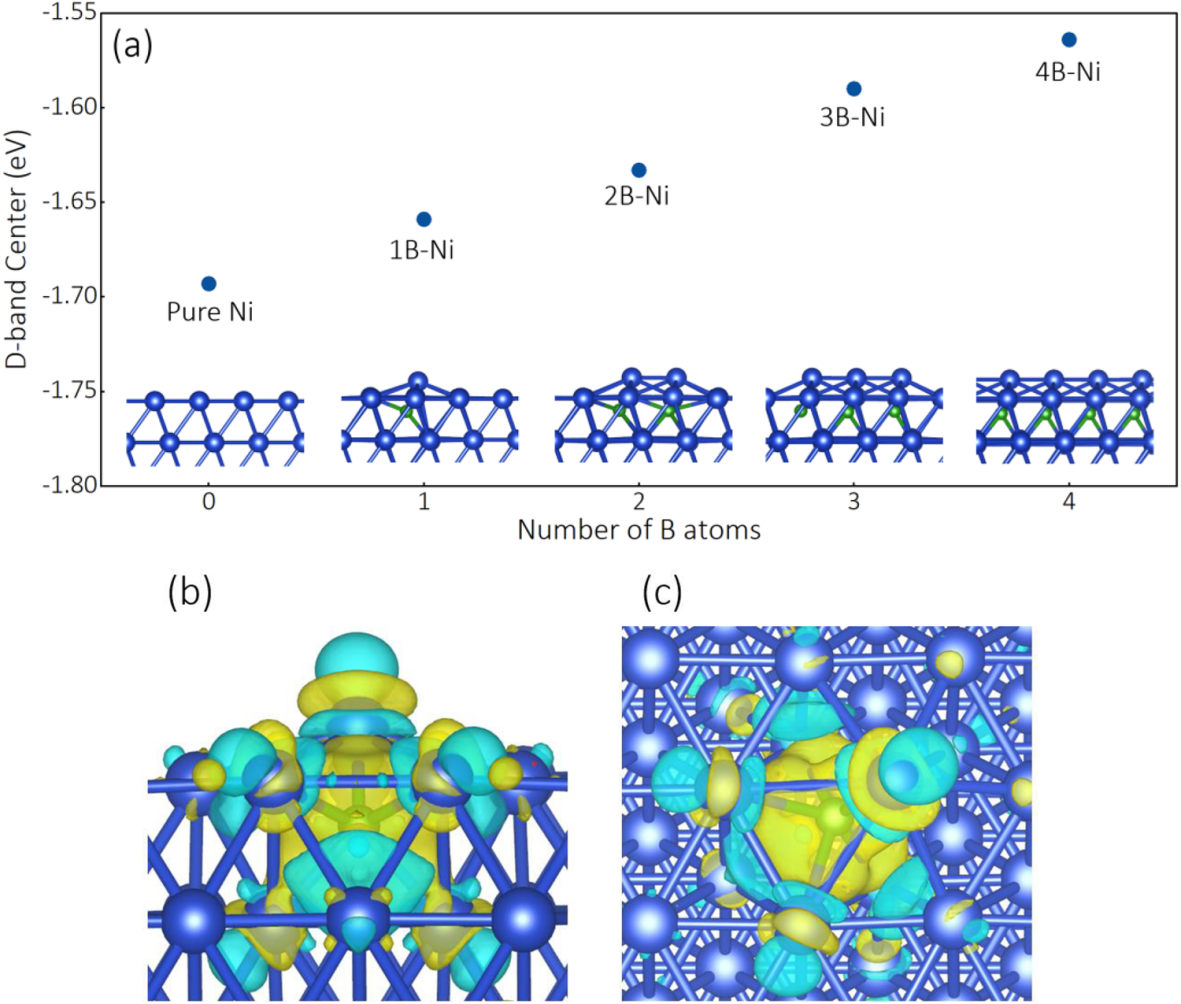
a) The d‐band center of Ni with different number of B atoms doping, b) side view, c) top view of the differential charge density distribution of 1B doped Ni(111) surface. Yellow and cyan contours represent electron accumulation and depletion, respectively.

The incorporation of boron atoms into Ni's interstitial sites induces two synergistic electronic modifications: lattice expansion effects and enhanced electronic interactions between neighboring atoms. Our DFT calculations (**Figure** **8**a) quantitatively demonstrate these effects through a 0.129 eV upshift of the d‐band center (from ‐1.693 eV for pure Ni to ‐1.564 eV for 4B‐doped Ni), which directly correlates with the experimentally observed enhancement in CH_4_ selectivity. This electronic modification strengthens the adsorption of CO intermediates—a critical factor confirmed by persistent CO signals in our in situ DRIFTS measurements (Figure 7a) throughout the catalytic reaction.^[^ ^48^ ^]^

Figure 8b,c demonstrate significant electron density accumulation in the subsurface region near boron atoms, confirming the thermodynamic preference for boron occupation of octahedral interstitial sites through strong host‐guest electronic interactions. This electron redistribution directly impacts the catalyst's surface reactivity, as evidenced by our experimental results showing a 2.4‐fold increase in CH_4_ yield (from 58.87 to 142.57 mmol g·h^−1^) when comparing thermal and photothermal conditions with 50% H_2_ (Figure 4a). The improved CO_2_ reduction performance can therefore be attributed to the synergistic combination of Ni d‐band upshifting and substantial electronic state mixing with interstitial boron atoms in subsurface layers, which creates more favorable energetics for both CO_2_ dissociation and subsequent hydrogenation steps

The projected density of states (PDOS) analysis of Ni 3d and C 2p orbitals (Figure 6) demonstrates that boron doping into subsurface Ni sites significantly enhances the orbital overlap between Ni 3d and C 2p states during CO adsorption compared to pristine Ni. This enhanced orbital overlap quantitatively explains our experimental observation of improved CH_4_ selectivity (increasing from 45.05% to 78.6% with higher H_2_ concentrations under thermal conditions, Figure 4a), as it facilitates stronger binding of CO on the boron‐doped Ni surface—a critical prerequisite for efficient hydrogenation to CH_4_.^[^ ^37^ ^]^ The computational results align perfectly with our in‐situ DRIFTS measurements, which showed persistent CO signals throughout the reaction period (Figure 7a), confirming that B‐doping facilitated CO adsorption without inhibiting subsequent hydrogenation steps

In addition to experimental findings, DFT calculations further validated the effect of boron doping and the competitive adsorption and dissociation of CO_2_ and H_2_O molecules on the catalyst surface. The adsorption properties of CO_2_ on Ni(111) and boron‐doped Ni(111) are summarized in Table S4 (Supporting Information), with the most stable adsorption sites identified as hcp sites near the boron doping location. The modeling of CO_2_ dissociation (*CO_2_ → *CO + *O) on these surfaces (Table S6, Supporting Information) indicates that boron‐doped Ni provides a more favorable environment for CO_2_ dissociation. The calculated free energy profile for this process under experimental conditions (310 °C, 1 bar) is illustrated in Figure 5a, demonstrating that boron‐doped Ni exhibits more favorable energetics for both CO_2_ adsorption and dissociation.

The adsorption properties of H_2_O on these surfaces (Table S5, Supporting Information) indicate that H_2_O molecules preferentially adsorb on the top sites of both surfaces. The dissociative energies of *OH, *O, and *H species at various adsorption sites are summarized in Tables S7 and S8 (Supporting Information). On the Ni(111) surface, *OH occupies the fcc site, while *H and *O tend to adsorb on fcc or hcp sites. Boron‐doped Ni shows enhanced performance for the *H_2_O → *OH + *H process, facilitating water dissociation.

The formation energies and adsorption sites of intermediates during CO_2_ reduction to CH_4_ on both surfaces are summarized in Tables S2 and S3 (Supporting Information). The calculated Gibbs free energy profile for the complete CO_2_ methanation pathway under experimental conditions (Figure 5d) provides valuable insights into the energetics of the reaction, with the adsorption configurations on boron‐doped Ni illustrated in Table S1 (Supporting Information).

The DFT‐calculated energy profiles in Figure 5 provide quantitative support for our experimental observations regarding reaction pathways and product selectivity. Specifically, the more favorable energetics for CO_2_ dissociation on B‐doped Ni (Figure 5a) directly explains the enhanced CO production observed in our experiments (61 mmol/g·h under photothermal conditions with 10% H_2_, Figure 3a). Furthermore, the calculated energy barriers for the complete CO_2_ methanation pathway (Figure 5c) reveal that B‐doping creates a more energetically favorable landscape for hydrogenation steps following CO formation, which aligns with our experimental finding that CH_4_ selectivity increases dramatically with higher H_2_ concentrations (from 45.05% with 10% H_2_ to 78.6% with 50% H_2_ under thermal conditions, Figure 4a). The computational results also explain why the addition of 5% H_2_O further enhanced CH_4_ selectivity to 80.21% (Figure 4c), as the DFT calculations show that H_2_O dissociation on B‐doped Ni provides additional hydrogen species that facilitate CO hydrogenation through lower energy pathways (Figure 5b).

### 2.6 Proposed Reaction Mechanism of CO_2_ Reduction with H_2_ and H_2_O

Previous studies have shown that the RWGS reaction typically proceeds through two primary pathways: direct CO_2_ dissociation (*CO_2_ → *CO + *O) or via carboxylate intermediates.^[^ ^21^, ^32^, ^49^, ^50^ ^]^ CO_2_ reduction with H_2_O has been reported to occur through either carboxylate or bicarbonate intermediates.^[^ ^37^ ^]^ However, the mechanistic role of H_2_O in RWGS reactions remains unexplored, particularly regarding its potential to alter reaction pathways.

The reaction pathways identified through our DFT calculations (Figure 5) provide the theoretical foundation for interpreting our experimental DRIFTS results. The calculated favorable energetics for direct CO_2_ dissociation on B‐doped Ni (‐0.21 eV activation barrier compared to ‐0.15 eV on pure Ni) quantitatively supports our observation that the RWGS reaction proceeds primarily through a direct dissociation pathway, as evidenced by the relatively weak *COOH signal (1541 cm^−1^) in our DRIFTS measurements (Figure 7a). Furthermore, the enhanced orbital overlap between Ni 3d and C 2p states upon B‐doping (Figure 6) explains the persistent CO signals detected throughout the reaction, as the stronger CO adsorption facilitates subsequent hydrogenation steps rather than CO desorption.

**Figure 9:**
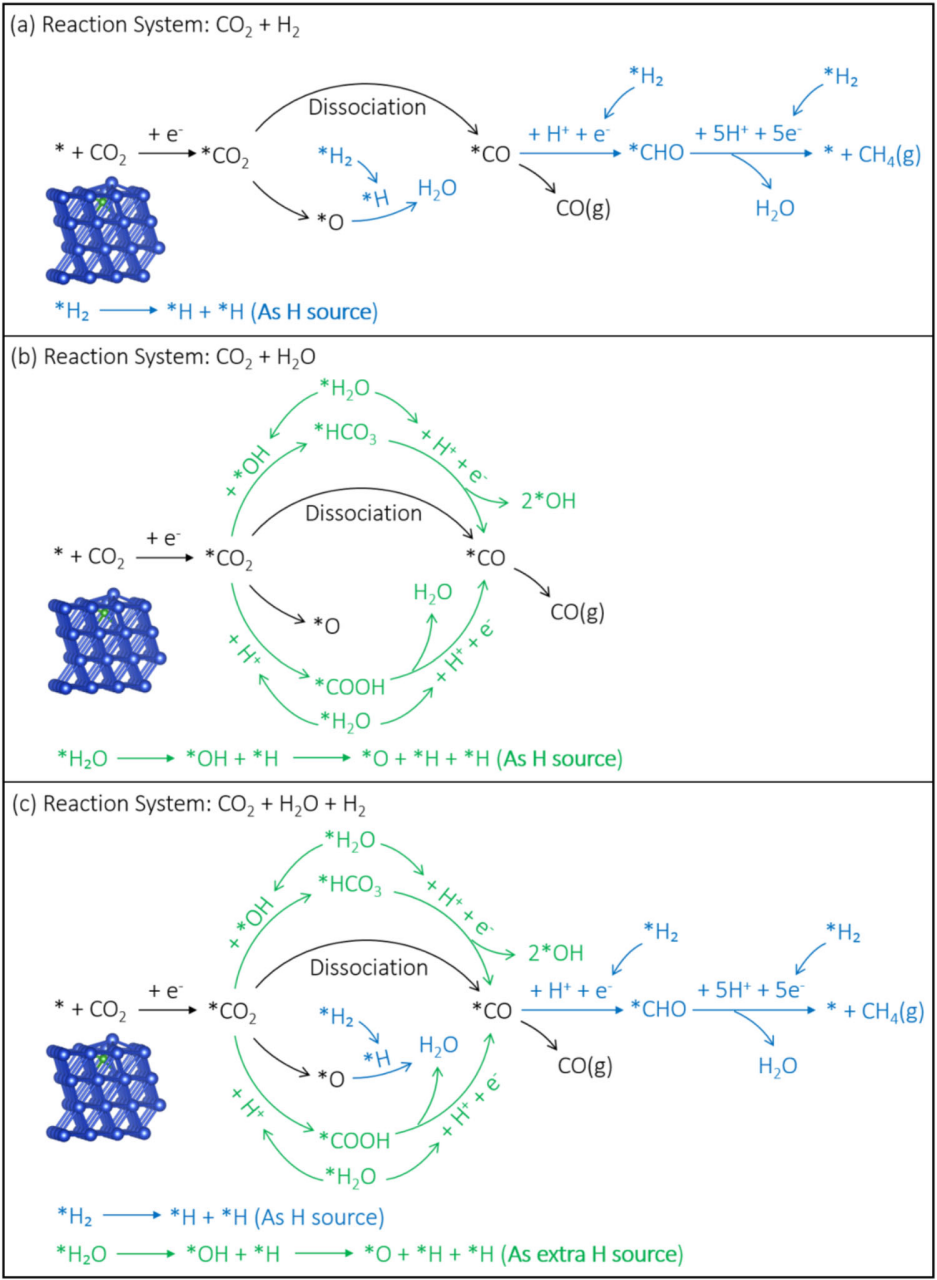
Proposed reaction mechanism of a) CO_2_ reduction with H_2_, b) CO_2_ reduction with H_2_O, c) CO_2_ reduction with H_2_ and H_2_O on B doped Ni.

To identify reaction intermediates during CO_2_ catalytic reduction on B doped Ni, in situ DRIFTS measurements were conducted. Figure 7a shows the results for CO_2_ reduction with H_2_ on boron‐doped Ni, revealing several surface species including monodentate carbonate (m‐CO_3_^2^^−^: 1373, 1505, and 1557 cm^−1^), bidentate carbonate (b‐CO_3_^2^^−^: 1337 cm^−1^ and 1652 cm^−1^), and H_2_O (1521 cm^−1^ and 1683 cm^−1^) species.^[^ ^29^, ^51^, ^52^, ^53^, ^54^ ^]^ CO was consistently detected throughout the entire 30‐minute measurement period. The characteristic band at 1541 cm^−1^, attributed to *COOH (a crucial intermediate in CO_2_ reduction to CO), exhibited relatively weak intensity. This observation suggests that the RWGS reaction on boron‐doped Ni predominantly occurs through a direct dissociation pathway, as clearly demonstrated in **Figure** **9**a.

DRIFTS was utilized to investigate reactive intermediates during CO_2_ reduction with H_2_O over boron‐doped Ni catalysts (Figure 7b). Comparative analysis with the CO_2_/H_2_ system revealed significantly enhanced intensities of *COOH (1541 cm^−1^) and HCO_3_^−^ (1410 cm^−1^) bands between 3–30 minutes, indicating two distinct reaction pathways: carboxylate formation through hydrogen transfer from dissociated H_2_O, and bicarbonate intermediate generation via hydroxide ion transfer. Interestingly, CO production was detected within the first minute of reaction despite the absence of characteristic *COOH and HCO_3_^−^ signals, unambiguously confirming the coexistence of a third, direct CO_2_ dissociation pathway. These results establish that CO generation in the CO_2_/H_2_O system proceeds through three competing mechanisms: carboxylate‐mediated, bicarbonate‐mediated, and direct dissociation routes (Figure 9b).

In the complete photothermal CO_2_/H_2_/H_2_O catalytic system, CO production similarly occurs through these three pathways. Importantly, hydrogen derived from H_2_O dissociation contributes to subsequent CO hydrogenation, thereby promoting CH_4_ formation and enhancing both methane yield and selectivity (Figure 9c).

The experimental results and mechanistic insights collectively reveal a delicate balance in the synergistic effects between H_2_ and H_2_O during CO_2_ reduction on boron‐doped Ni. At optimal concentrations (50% H_2_, 5% H_2_O), these two hydrogen sources work cooperatively through complementary mechanisms: H_2_ provides the primary reducing power for CO hydrogenation as evidenced by the strong correlation between H_2_ concentration and CH_4_ yield (Figure 4a), while H_2_O enables additional CO_2_ activation pathways through carboxylate and bicarbonate intermediates as confirmed by our in situ DRIFTS measurements (Figure 7b). The enhanced CH_4_ selectivity (80.21%) achieved under these conditions can be attributed to three synergistic factors: (1) B‐doping facilitated CO adsorption through d‐band center upshifting (‐1.564 eV versus ‐1.693 eV for pure Ni, Figure 8a), creating an optimal binding strength for subsequent hydrogenation; (2) sufficient H_2_ supply to drive the eight‐electron reduction process from CO_2_ to CH_4_; and (3) H_2_O‐derived hydrogen species that provide additional reduction capacity while simultaneously activating alternative CO_2_ conversion pathways. This mechanistic understanding explains why the optimal performance is achieved at moderate H_2_O concentrations (5%), where synergistic effects are maximized before competitive adsorption becomes dominant.

## 3 Conclusion

This study demonstrates significantly enhanced CO_2_ reduction to CH_4_ using boron‐doped Ni catalysts under photothermal conditions. Increasing H_2_ concentration from 10% to 50% improved CH_4_ yield by 2.6‐fold under thermal conditions and 4.3‐fold under photothermal conditions. The strategic addition of 5% water vapor further boosted CH_4_ productivity to 195.85 mmol g·h^−1^ with exceptional selectivity (80.21%), representing one of the highest reported values for Ni‐based catalysts.

Our mechanistic investigations revealed that CO_2_ reduction proceeds through three competing pathways: direct dissociation, carboxylate‐mediated, and bicarbonate‐mediated routes. Boron doping upshifts the Ni d‐band center from ‐1.693 eV to ‐1.564 eV, enhancing CO adsorption through greater orbital overlap between Ni 3d and C 2p states, which facilitates subsequent hydrogenation to CH_4_.

We identified a synergistic relationship between H_2_ and H_2_O, where H_2_O provides additional hydrogen species through dissociation while enabling alternative CO_2_ activation pathways that complement the primary H_2_‐driven reduction process. This explains why optimal performance occurs at moderate H_2_O concentrations (5%), before competitive adsorption becomes dominant.

These findings advance our understanding of catalyst electronic structure‐performance relationships and provide a foundation for developing more efficient CO_2_ conversion technologies for renewable fuel production and climate change mitigation.

## Conflict of Interest

The authors declare no conflict of interest.

## Supporting information



Supporting Information

## Data Availability

The data that support the findings of this study are available from the corresponding author upon reasonable request.

## References

[1] S. I. Seneviratne , M. G. Donat , A. J. Pitman , R. Knutti , R. L. Wilby , Nature 2016, 529, 477. 26789252 10.1038/nature16542

[2] N. Antonovsky , S. Gleizer , E. Noor , Y. Zohar , E. Herz , U. Barenholz , L. Zelcbuch , S. Amram , A. Wides , N. Tepper , Cell 2016, 166, 115. 27345370 10.1016/j.cell.2016.05.064 PMC4930481

[3] J. Klankermayer , W. Leitner , Science 2015, 350, 629. 26542554 10.1126/science.aac7997

[4] D. Xu , Y. Wang , M. Ding , X. Hong , G. Liu , S. C. E. Tsang , Chem 2021, 7, 849.

[5] M. Aziz , A. Jalil , S. Triwahyono , A. Ahmad , Green Chem. 2015, 17, 2647.

[6] Z. Wang , X. Mao , P. Chen , M. Xiao , S. A. Monny , S. Wang , M. Konarova , A. Du , L. Wang , Angew. Chem.‐Int. Ed. 2019, 131, 1042. 10.1002/anie.201810583 30417505

[7] M. Cai , Z. Wu , Z. Li , L. Wang , W. Sun , A. A. Tountas , C. Li , S. Wang , K. Feng , A.‐B. Xu , Nat. Energy 2021, 6, 807.

[8] U. Ulmer , T. Dingle , P. N. Duchesne , R. H. Morris , A. Tavasoli , T. Wood , G. A. Ozin , Nat. Commun. 2019, 10, 3169. 31320620 10.1038/s41467-019-10996-2 PMC6639413

[9] C. Song , Z. Wang , Z. Yin , D. Xiao , D. Ma , Chem Catal. 2022, 2, 52.

[10] M. Ghoussoub , M. Xia , P. N. Duchesne , D. Segal , G. Ozin , Energy Environ. Sci. 2019, 12, 1122.

[11] X. Meng , T. Wang , L. Liu , S. Ouyang , P. Li , H. Hu , T. Kako , H. Iwai , A. Tanaka , J. Ye , Angew. Chem.‐Int. Ed. 2014, 53, 11478. 10.1002/anie.201404953 25044684

[12] X. Sun , S. Jiang , H. Huang , H. Li , B. Jia , T. Ma , Angew. Chem.‐Int. Ed. 2022, 134, 202204880. 10.1002/anie.202204880 PMC9400894 35471594

[13] K. Peng , J. Ye , H. Wang , H. Song , B. Deng , S. Song , Y. Wang , L. Zuo , J. Ye , Appl. Catal., B 2023, 324, 122262.

[14] R.‐P. Ye , W. Gong , Z. Sun , Q. Sheng , X. Shi , T. Wang , Y. Yao , J. J. Razink , L. Lin , Z. Zhou , Energy 2019, 188, 116059.

[15] W. Jin , Y. Wang , T. Liu , C. Ding , H. Guo , Appl. Surf. Sci. 2022, 599, 154024.

[16] X. Liu , L. Sun , W.‐Q. Deng , J. Phys. Chem. C 2018, 122, 8306.

[17] A. Vita , C. Italiano , L. Pino , P. Frontera , M. Ferraro , V. Antonucci , Appl. Catal., B 2018, 226, 384.

[18] H. Liu , T. D. Dao , L. Liu , X. Meng , T. Nagao , J. Ye , Appl. Catal., B 2017, 209, 183.

[19] G. Zhou , H. Liu , K. Cui , A. Jia , G. Hu , Z. Jiao , Y. Liu , X. Zhang , Appl. Surf. Sci. 2016, 383, 248.

[20] M. Cai , Z. Wu , Z. Li , L. Wang , W. Sun , A. A. Tountas , C. Li , S. Wang , K. Feng , A.‐B. Xu , Nat. Energy 2021, 6, 807.

[21] S. Wang , D. Zhang , W. Wang , J. Zhong , K. Feng , Z. Wu , B. Du , J. He , Z. Li , L. He , Nat. Commun. 2022, 13, 5305. 36085305 10.1038/s41467-022-33029-x PMC9463155

[22] F. Raziq , C. Feng , M. Hu , S. Zuo , M. Z. Rahman , Y. Yan , Q.‐H. Li , J. Gascon , H. Zhang , J. Am. Chem. Soc. 2024, 146, 21008. 38869376 10.1021/jacs.4c05873

[23] S. Mo , S. Li , J. Zhou , X. Zhao , H. Zhao , X. Zhou , Y. Fan , Z. Zhu , B. Li , Q. Xie , ACS Catal. 2025, 15, 2796.

[24] X. Yan , M. Cao , S. Li , P. N. Duchesne , W. Sun , C. Mao , R. Song , Z. Lu , X. Chen , W. Qian , J. Am. Chem. Soc. 2023, 145, 27358. 38052446 10.1021/jacs.3c07668

[25] Y. Li , F. Meng , Q. Wu , D. Yuan , H. Wang , B. Liu , J. Wang , X. San , L. Gu , Q. Meng , Sci. Adv. 2024, 10, adn5098. 10.1126/sciadv.adn5098 PMC11100559 38758784

[26] T. Koretsune , S. Saito , Phys. Rev. B—Condens. Matter Mater. Phys. 2008, 77, 165417.

[27] S. Agnoli , M. Favaro , J. Mater. Chem. A 2016, 4, 5002.

[28] T. Chen , C. Foo , S. C. E. Tsang , Chem. Sci. 2021, 12, 517.

[29] H. Long , D. Gao , P. Wang , X. Wang , F. Chen , H. Yu , Appl. Catal., B 2024, 340, 123270.

[30] F. Yang , P. Han , N. Yao , G. Cheng , S. Chen , W. Luo , Chem. Sci. 2020, 11, 12118. 34094426 10.1039/d0sc03917a PMC8162945

[31] M. Tahir , N. S. Amin , Energy Convers. Manage. 2013, 76, 194.

[32] C. Vogt , E. Groeneveld , G. Kamsma , M. Nachtegaal , L. Lu , C. J. Kiely , P. H. Berben , F. Meirer , B. M. Weckhuysen , Nat. Catal. 2018, 1, 127.

[33] J. F. Simons , T. J. de Heer , R. C. van de Poll , V. Muravev , N. Kosinov , E. J. Hensen , J. Am. Chem. Soc. 2023, 145, 20289. 37677099 10.1021/jacs.3c04284 PMC10515628

[34] J. A. H. Lalinde , P. Roongruangsree , J. Ilsemann , M. Baeumer , J. Kopyscinski , Chem. Eng. J. 2020, 390, 124629.

[35] M. Gu , S. Dai , R. Qiu , M. E. Ford , C. Cao , I. E. Wachs , M. Zhu , ACS Catal. 2021, 11, 12609.

[36] J. K. Prabhakar , P. A. Apte , G. Deo , Chem. Eng. J. 2023, 471, 144252.

[37] Y. Zhou , F. Che , M. Liu , C. Zou , Z. Liang , P. De Luna , H. Yuan , J. Li , Z. Wang , H. Xie , Nat. Chem. 2018, 10, 974. 30013194 10.1038/s41557-018-0092-x

[38] C. W. A. Chan , A. H. Mahadi , M. M.‐J. Li , E. C. Corbos , C. Tang , G. Jones , W. C. H. Kuo , J. Cookson , C. M. Brown , P. T. Bishop , Nat. Commun. 2014, 5, 5787. 25523894 10.1038/ncomms6787

[39] B. Ravel , M. Newville , A. R. T. E. M. I. S. Athena , Synchrotron Radiat. 2005, 12, 537. 10.1107/S0909049505012719 15968136

[40] B. Su , S. B. Wang , W. D. Xing , K. L. Liu , S. F. Hung , X. Chen , Y. X. Fang , G. G. Zhang , H. B. Zhang , X. C. Wang , Angew. Chem.‐Int. Ed. 2025.

[41] J. L. Li , J. W. Zhao , S. B. Wang , K. S. Peng , B. Su , K. L. Liu , S. F. Hung , M. R. Huang , G. G. Zhang , H. B. Zhang , X. C. Wang , J. Am. Chem. Soc. 2025, 147, 14705. 40239055 10.1021/jacs.5c03098

[42] J. Wang , S. Li , J. Zhao , K. Liu , B. Jiang , H. Li , Appl. Catal. B: Environ. 2024, 352, 124045.

[43] T. Dong , X. Liu , Z. Tang , H. Yuan , D. Jiang , Y. Wang , Z. Liu , X. Zhang , S. Huang , H. Liu , Appl. Catal., B 2023, 326, 122176.

[44] Y. Tang , T. Zhao , H. Han , Z. Yang , J. Liu , X. Wen , F. Wang , Adv. Sci. 2023, 10, 2300122. 10.1002/advs.202300122 PMC10214215 36932051

[45] X. Yu , X. Ding , Y. Yao , W. Gao , C. Wang , C. Wu , C. Wu , B. Wang , L. Wang , Z. Zou , Adv. Mater. 2024, 36, 2312942. 10.1002/adma.202312942 38354694

[46] L. Wang , Y. Dong , T. Yan , Z. Hu , F. M. Ali , D. M. Meira , P. N. Duchesne , J. Y. Y. Loh , C. Qiu , E. E. Storey , Nat. Commun. 2020, 11, 2432. 32415078 10.1038/s41467-020-16336-z PMC7229034

[47] Z. Li , C. Mao , Q. Pei , P. N. Duchesne , T. He , M. Xia , J. Wang , L. Wang , R. Song , F. M. Ali , Nat. Commun. 2022, 13, 7205. 36418855 10.1038/s41467-022-34798-1 PMC9684568

[48] A. Nilsson , L. G. Pettersson , J. Norskov , in Chemical Bonding at Surfaces and Interfaces, Elsevier, Amsterdam, Netherlands 2011.

[49] S. Kattel , P. Liu , J. G. Chen , J. Am. Chem. Soc. 2017, 139, 9739. 28650651 10.1021/jacs.7b05362

[50] X. Yan , W. Sun , L. Fan , P. N. Duchesne , W. Wang , C. Kübel , D. Wang , S. G. H. Kumar , Y. F. Li , A. Tavasoli , Nat. Commun. 2019, 10, 2608. 31197151 10.1038/s41467-019-10464-x PMC6565710

[51] J. Xu , Z. Ju , W. Zhang , Y. Pan , J. Zhu , J. Mao , X. Zheng , H. Fu , M. Yuan , H. Chen , Angew. Chem.‐Int. Ed. 2021, 133, 8787. 10.1002/anie.202017041 33470491

[52] Y. Shi , G. Zhan , H. Li , X. Wang , X. Liu , L. Shi , K. Wei , C. Ling , Z. Li , H. Wang , Adv. Mater. 2021, 33, 2100143. 10.1002/adma.202100143 34331321

[53] J. Wu , X. Li , W. Shi , P. Ling , Y. Sun , X. Jiao , S. Gao , L. Liang , J. Xu , W. Yan , Angew. Chem.‐Int. Ed. 2018, 130, 8855. 10.1002/anie.201803514 29761617

[54] Y. Ren , Y. Si , M. Du , C. You , C. Zhang , Y. H. Zhu , Z. Sun , K. Huang , M. Liu , L. Duan , Angew. Chem.‐Int. Ed. 2024, 136, 202410474. 10.1002/anie.202410474 39087314

