# Supplementary material for: Water Vapor‐Enhanced Selective Production of Methane During Photothermal CO2 Reduction: Mechanistic Insights Into Boron‐Doped Nickel Catalysts
Source: Adv Sci (Weinh). 2025 Nov 16;13(6):e19611. doi: 10.1002/advs.202519611 (PMC12866817; doi:10.1002/advs.202519611)
Supplement: Supplementary file 1 — Supporting Information [file ADVS-13-e19611-s001.docx]

**Water Vapor-Enhanced Selective Production of Methane During Photothermal CO_2_ Reduction: Mechanistic Insights into Boron-Doped Nickel Catalysts**

Cong Wan^1, 2^, Yiming Li^1, 2^, Min Liu^1, 2^, Honglei Zhang^1, 2^, Jianwen Zhang^1, 2^, Chengrui Xie^1, 2^_,_ Huiwen Zhu^1, 2^, Zijun Yan^1, 2^, Tao Wu^1, 2*^

1 Department of Chemical and Environmental Engineering, University of Nottingham, Ningbo China, Ningbo 315100, China

2 Municipal Key Laboratory of Clean Energy Technologies of Ningbo, University of Nottingham Ningbo China, Ningbo 315100, China

Corresponding author:

Tao Wu, tao.wu@nottingham.edu.cn

1. **Experimental Section**
   1. **Material Preparation**

Boron-doped Ni catalysts were synthesized using a facile reduction method. In a typical synthesis, 2.25 mmol of NiCl₂·6H₂O was dissolved in 50 mL of deionized water (solution A), while 12.5 mmol of NaBH₄ was dissolved in 50 mL of deionized water (solution B). Solution B was added dropwise to solution A with continuous stirring under ambient conditions. During this process, vigorous bubbling occurred with the formation of black precipitates. After 30 minutes of stirring, the solution was washed with deionized water and ethanol, and the resulting precursor was freeze-dried overnight to obtain an amorphous B-Ni precursor. This precursor was then calcined at 310°C for 2 hours under different atmospheres: CO₂ with 10% H₂ to form B₂O₃/Ni, or CO₂ with 50% H₂ to produce boron-doped Ni catalysts.

- 1. **CO_2_ Reduction Tests**

All photothermal reduction experiments were conducted using a hollow quartz tube (6 mm diameter) with a solid quartz tube (5 mm diameter) fixed in the center. For each experiment, 10 mg of catalyst was compacted into a 0.5 mm thick bed.

Photo-induced Illumination was provided by two 300 W xenon arc lamps equipped with an AM1.5G optical filter and plano-convex lens, delivering a light intensity of 2000 mW/cm². The surface temperature of catalyst during illumination was tested using an infrared thermal imaging camera (Testo 868).

Thermally assisted photothermal reduction was investigated using a tube furnace fitted with a quartz window (CEL-GPPCH, Beijing China Education AU-LIGHT Co, Ltd.). Illumination was provided by two 300 W xenon arc lamps (320-780 nm wavelength range) with a combined light intensity of 800 mW/cm². External heating was supplied by the tube furnace.

Prior to each experiment, the system was purged with high-purity argon (20 mL/min) for 30 minutes to eliminate ambient air. Subsequently, a gas flow of 10 mL/min was introduced for the reduction reaction. H₂ content was controlled by adjusting the flow rates of H₂ and CO₂. For experiments with water vapor, CO₂ was bubbled through a temperature-controlled water bath to achieve the desired H₂O content. All experiments were performed at atmospheric pressure.

Gas product composition was continuously monitored at 8-minute intervals using an Agilent GC-8890 gas chromatograph equipped with a thermal conductivity detector (TCD) for hydrogen (H₂) analysis and two flame ionization detectors (FIDs) for simultaneous quantification of carbon monoxide (CO), methane (CH₄), and ethane (C₂H₆), enabling comprehensive tracking of reaction products and byproducts throughout the catalytic process.

- 1. **Characterization Techniques**

Structural and chemical characterization was performed using complementary analytical techniques: X-ray diffraction (XRD) patterns were collected on a Bruker D8 Advance diffractometer operating in θ-2θ geometry (20-80° range) and refined using MDI Jade 6 software. Morphological analysis was conducted using a ZEISS Merlin 360 scanning electron microscope (SEM), while transmission electron microscopy (TEM) investigations, including high-resolution imaging (HRTEM) and energy-dispersive X-ray spectroscopy (EDS) mapping, were performed on a JEOL JEM-2100F field-emission microscope operated at 200 kV. Surface chemical states were analyzed by X-ray photoelectron spectroscopy (XPS) using a Kratos Axis Supra+ system with monochromatic Al Kα radiation (1486.6 eV). Local electronic structure and coordination environment were probed by X-ray absorption spectroscopy (XAS) at the Ni K-edge (8333 eV) using the Rapid XAFS 2M beamline (Anhui Absorption Spectroscopy Analysis Instrument Co., Ltd.), with spectra collected in transmission mode using a silicon drift detector and referenced to Ni foil. All XAS data processing was performed using the Demeter software package (Athena/Artemis modules). [39]

In situ diffuse reflectance infrared Fourier transform spectroscopy (DRIFTS) was conducted using a Thermo Fisher Scientific Nicolet 6700 spectrometer equipped with a liquid nitrogen-cooled mercury-cadmium-tellurium (MCT) detector and a high-temperature reaction chamber. For each measurement, approximately 30 mg of sample powder was loaded into the sample cup of the high-temperature cell. The sample was initially purged for 30 minutes at 300°C with N₂ (20 mL/min) to remove pre-adsorbed species, and a background spectrum was recorded. The gas flow was then switched to the reaction mixture (CO₂/H₂ or CO₂/H₂O at 20 mL/min, with CO₂:H₂ = 9:1). Spectra were recorded at 1, 3, 5, 10, 15, 20, 25, and 30 minutes to monitor the evolution of surface species.

- 1. **Computational Method**

Density functional theory (DFT) calculations were performed using the Vienna Ab initio Simulation Package (VASP) with a nanoscale Ni(111) model constructed as a 3 × 3 supercell containing three atomic layers under periodic boundary conditions in the x-y plane, incorporating a 15 Å vacuum layer along the z-axis to eliminate periodic image interactions. Systematic evaluation of boron doping sites revealed the subsurface octahedral position as the most stable configuration. The calculations employed a plane-wave energy cutoff of 400 eV with Brillouin zone sampling using a 3 × 3 × 1 Monkhorst-Pack k-point grid, treating exchange-correlation interactions through the generalized gradient approximation (GGA) with the Perdew-Burke-Ernzerhof (PBE) functional, while core electrons and ionic effects were accounted for using the projector augmented wave (PAW) method with explicit spin polarization for Ni atoms.

Structural optimization was carried out using a conjugate gradient algorithm, with iterations continuing until the forces on each atom were reduced to below 0.02 eV/Å. The adsorption energies of CO₂ and H₂O on the boron-doped Ni surface were calculated using the following equation:

$E_{ads}=E_{adsorbate+surface}-E_{adsorbate}-E_{surface}$ (1)

Where *E_adsorbate+surface_*, *E_adsorbate_*, and *E_surface_* are the energy of the adsorbed system, the gas-phase molecule, and the bare surface, respectively. Similarly, the dissociative adsorption energy for CO* and O*, OH* and H*, O* and H* is calculated by:

${\Delta E}_{chem}=E_{dissociated adsorbate+surface}-E_{adsorbate}-E_{surface}$ (2)

For the phase changed Gibbs free energy, such as CO adsorption on the Ni surface at 310℃ and 1 atm, it is calculated by:

$\Delta G_{CO}\left( T,P^{0} \right)=E_{ads}+\Delta H^{0}\left( T \right)-T\Delta S^{0}(T,P^{0})$ (3)

$\Delta H^{0}\left( T \right)=H_{trans}^{A}+H_{rot}^{A}+H_{vib}^{A}-H_{vib}^{A*}$ (4)

$\Delta S^{0}\left( T \right)=S_{trans}^{A}+S_{rot}^{A}+S_{vib}^{A}-S_{vib}^{A*}$ (5)

The enthalpy (H) and entropy (S) terms account for: translational (trans), rotational (rot), and vibrational (vib) contributions; gas-phase (A) versus adsorbed (A*) state differences and temperature-dependent thermodynamic corrections.

**1.5. Statistical Analysis**

1.5.1. Data Pre-processing

- All catalytic performance data were collected from at least three independent experimental replicates;
- Catalyst characterization data were obtained from multiple regions of each sample to ensure representativeness;
- No data points were excluded as outliers unless clearly identified as experimental artifacts.

1.5.2. Data Presentation

- Equation (6) was applied to calculate the yield of CH_4_(Yield_CH4_) or CO(Yield_CO_):

${Yield}_{{CH}_{4}}\left( \mathrm{or}{Yield}_{CO} \right)=\frac{n_{{CH}_{4}}(or n_{CO})}{weight of catalyst}\times60 (mmol/g\cdot h)$ (6)

The selectivity was determined by equation (7):

$S_{{CH}_{4}}\left( or S_{CO} \right)= \frac{C_{{CH}_{4}}(or C_{CO})}{C_{{CH}_{4}}+C_{CO}}$ (7)

Where, $n_{{CH}_{4}}\left( or n_{CO} \right)= \frac{flow rate \times C_{{CH}_{4}}(or C_{CO})}{22.4}$, *flow rate* is the total gas flow rate (ml/min), *C_CH4_*(or *C_co_*) signs the respective concentration (vol.%) of the corresponding substance detected by online GC.

1.5.3. Sample Size

- Catalytic performance tests: n ≥ 3 independent experiments;
- Material characterization: n ≥ 5 different regions per sample;
- DFT calculations: multiple initial configurations were tested for each model.

1.5.4. Software

- Origin 2022 was used for statistical analysis and graph preparation;
- All DFT-related statistics were processed within the VASP package environment;
- X-ray diffraction (XRD) patterns were refined using MDI Jade 6 software;
- All XAS data processing was performed using the Demeter software package (Athena/Artemis modules).


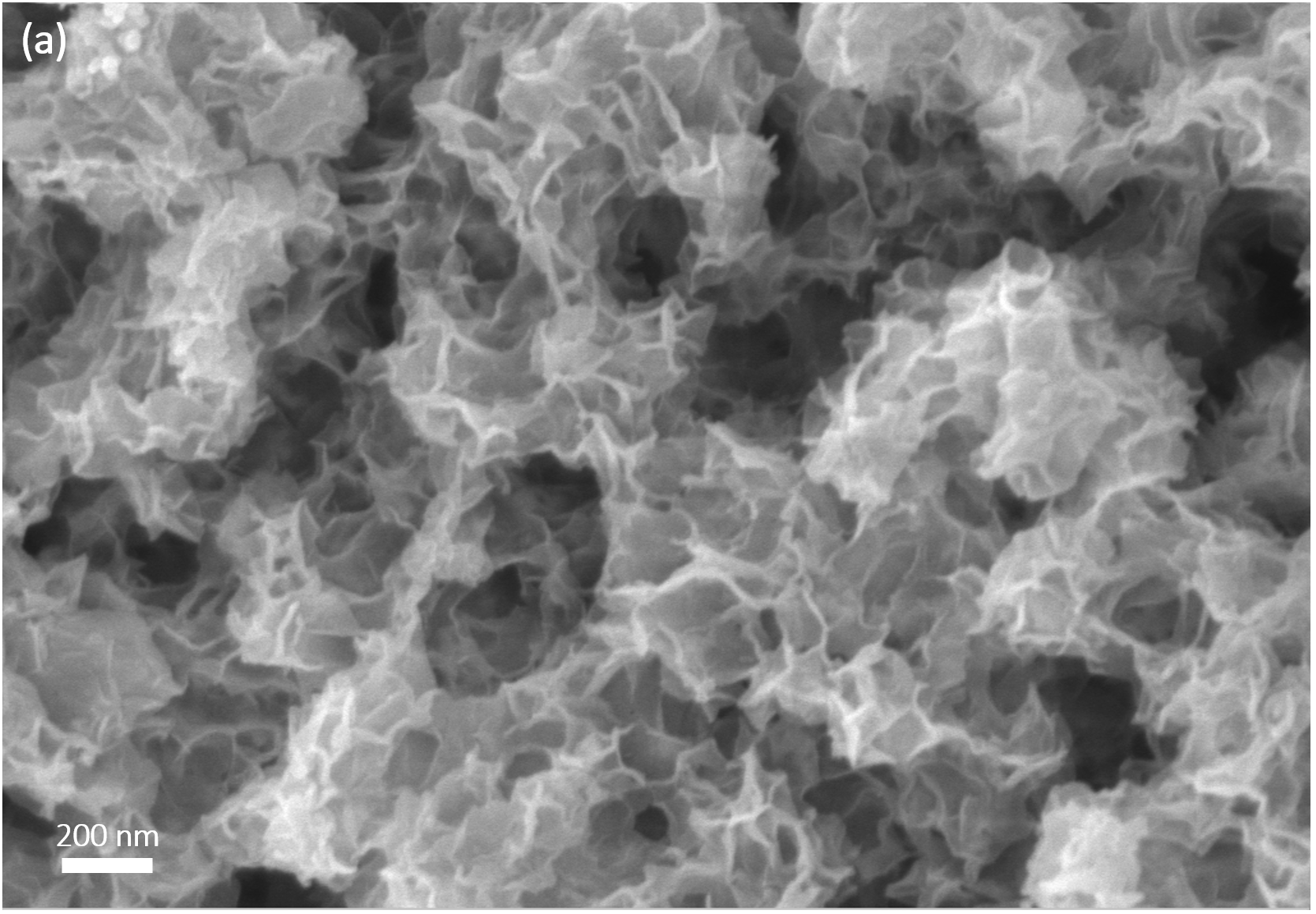


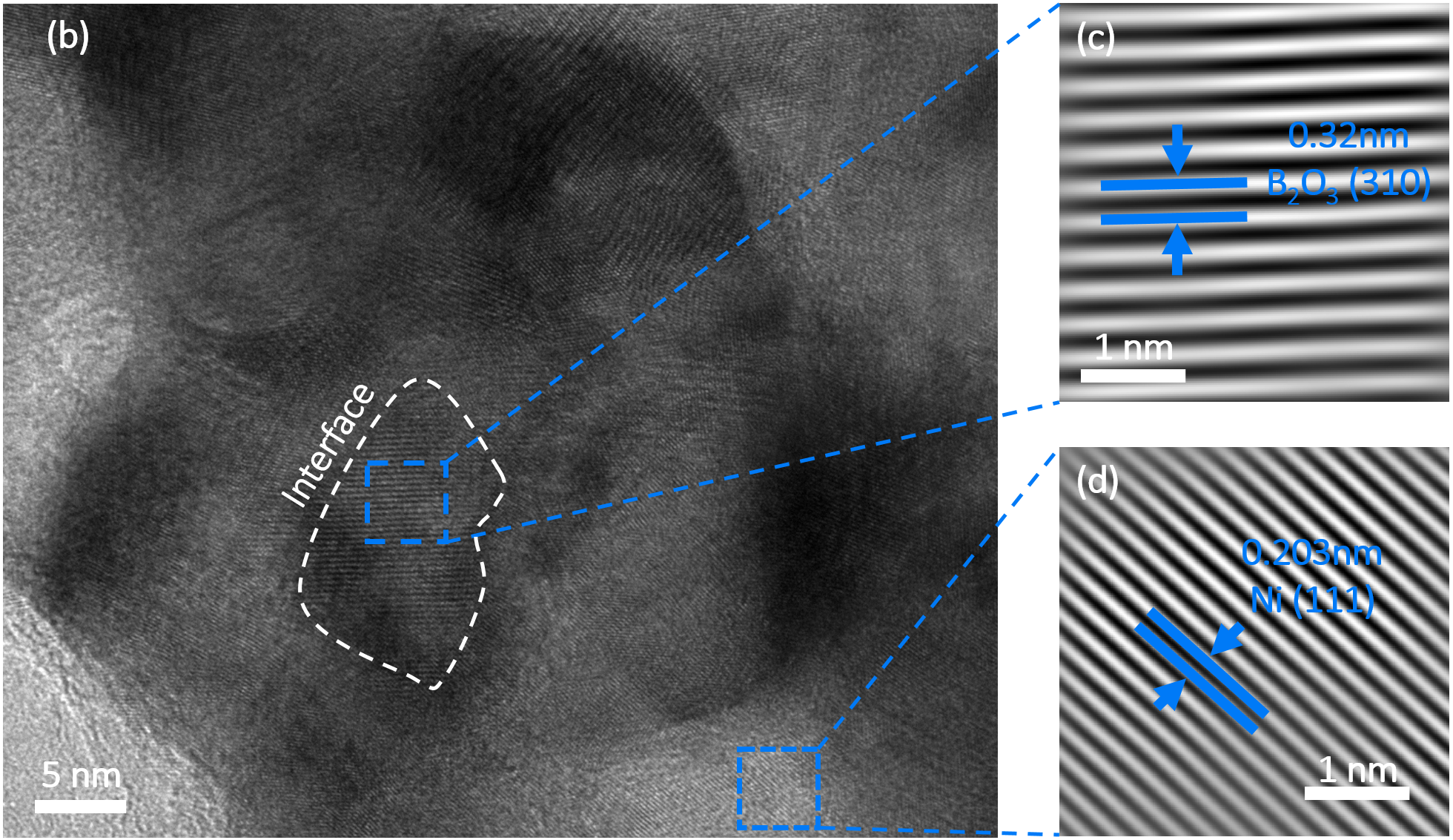


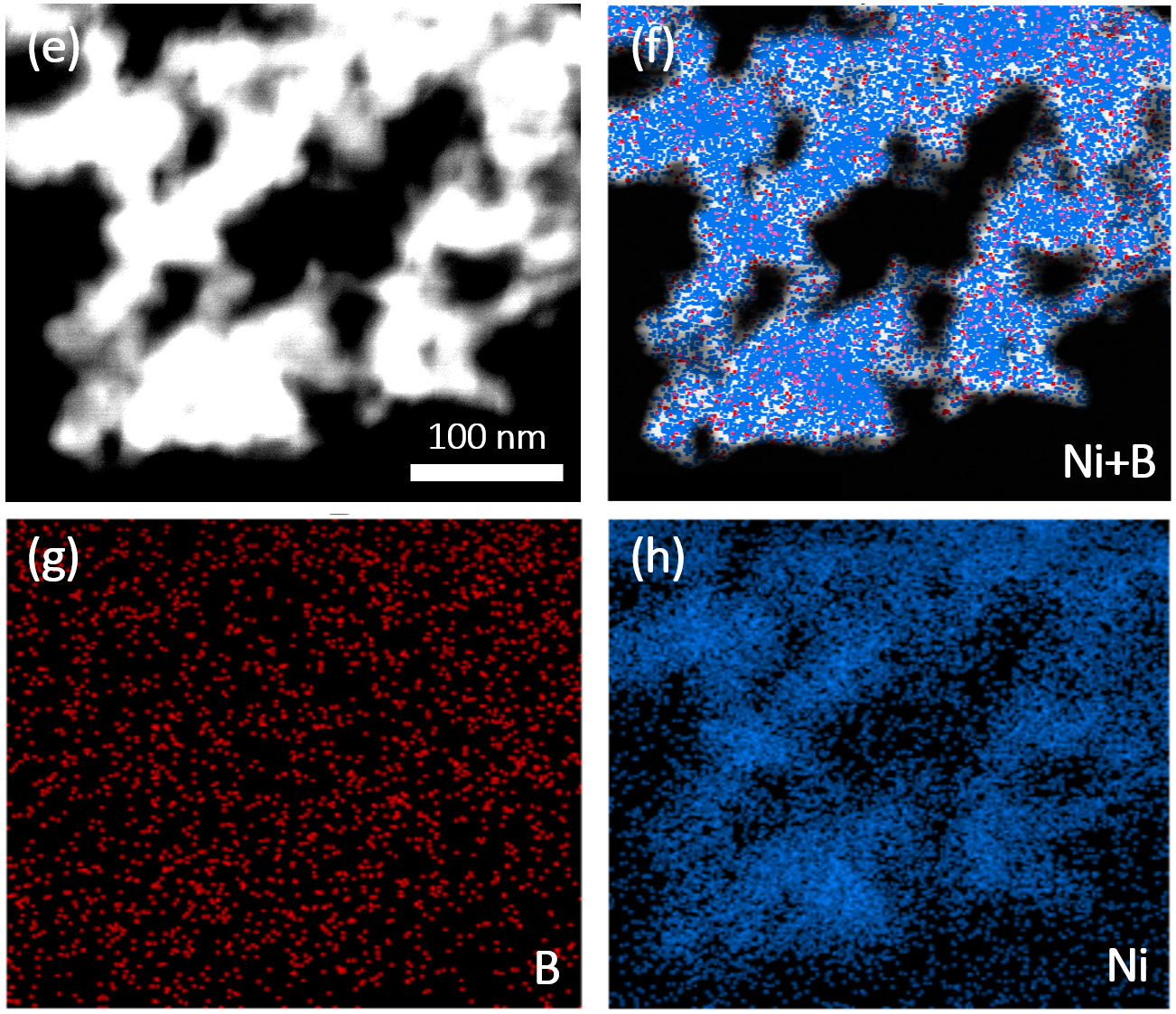


**Fig.S1 (a) SEM image, (b-d) HRTEM image, and (e-h) HAADF image and the element mapping of B and Ni of B_2_O_3_/Ni.**


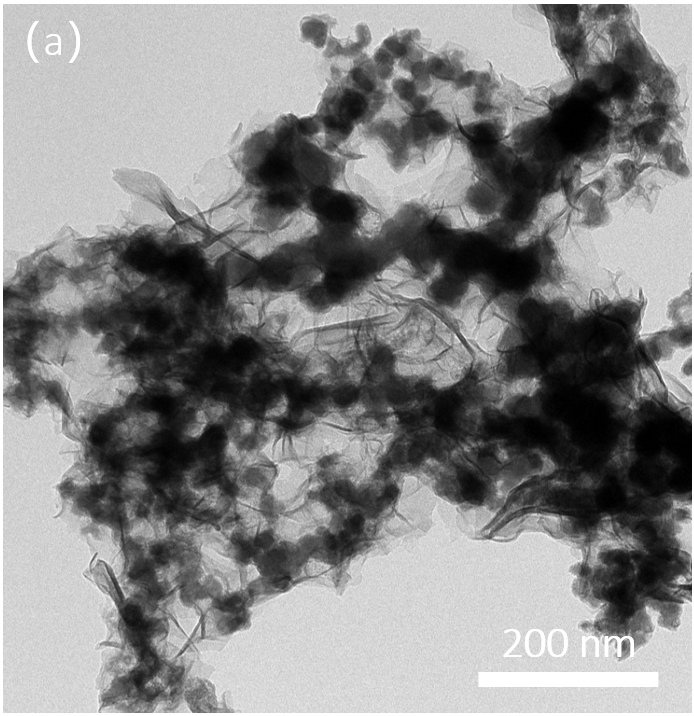

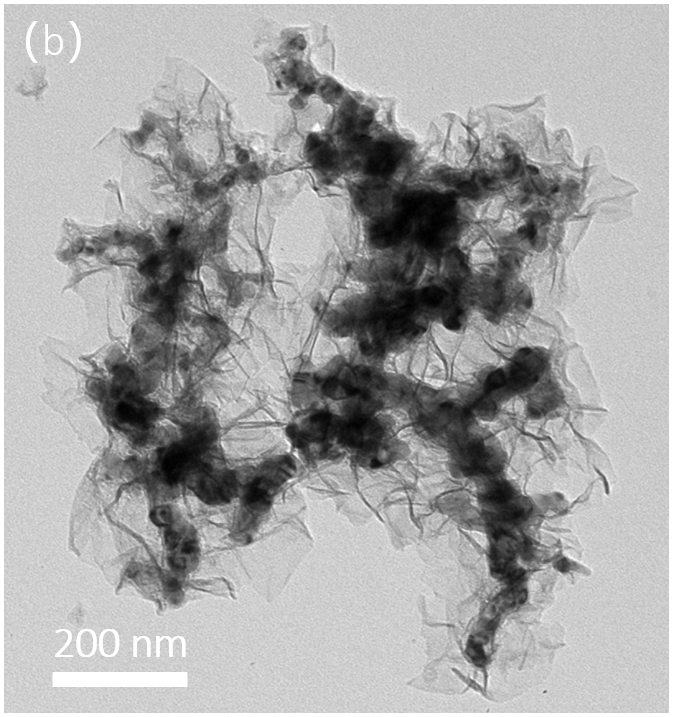


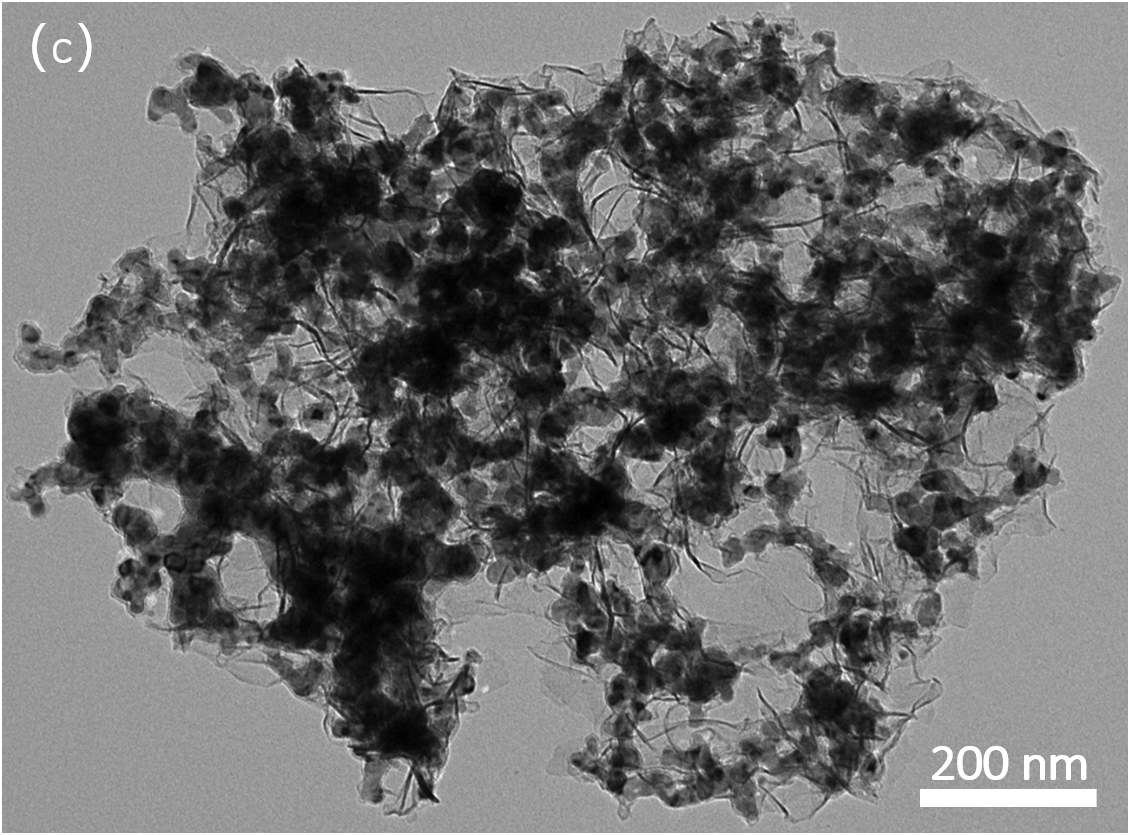


**Fig.S2 TEM images of (a) Amorphous B-Ni precursor, (b) B_2_O_3_/Ni, (c) B doped Ni.**


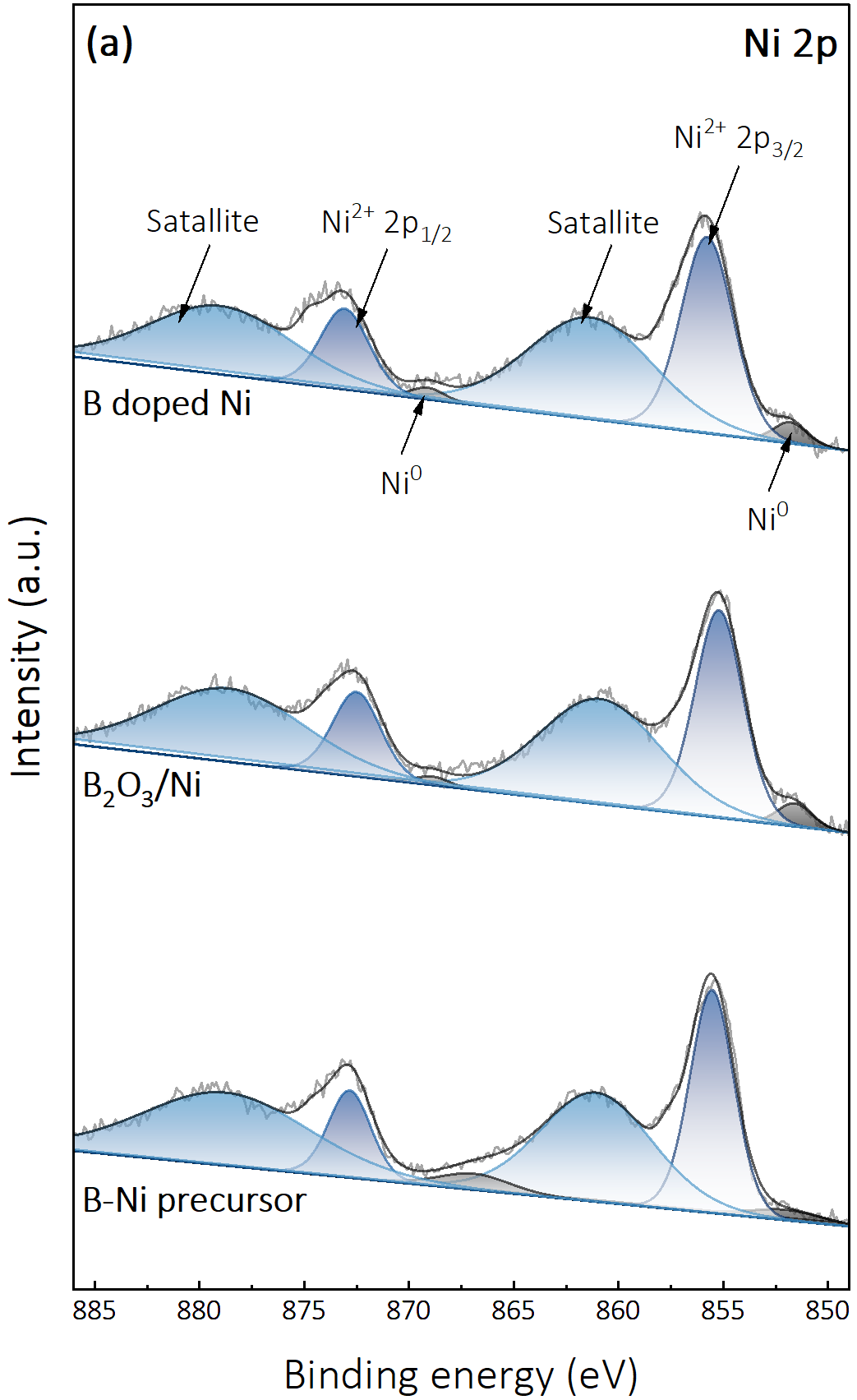

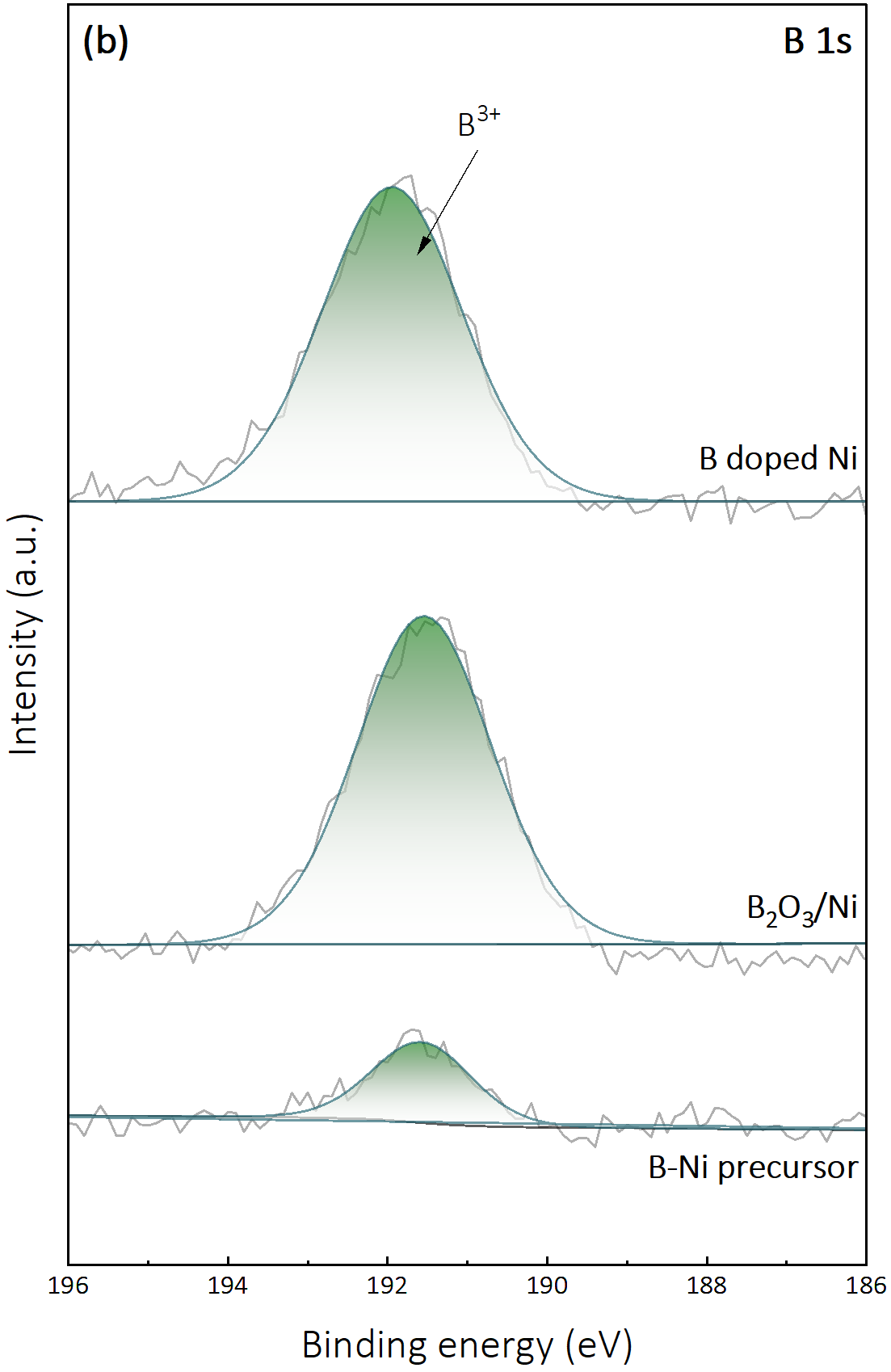


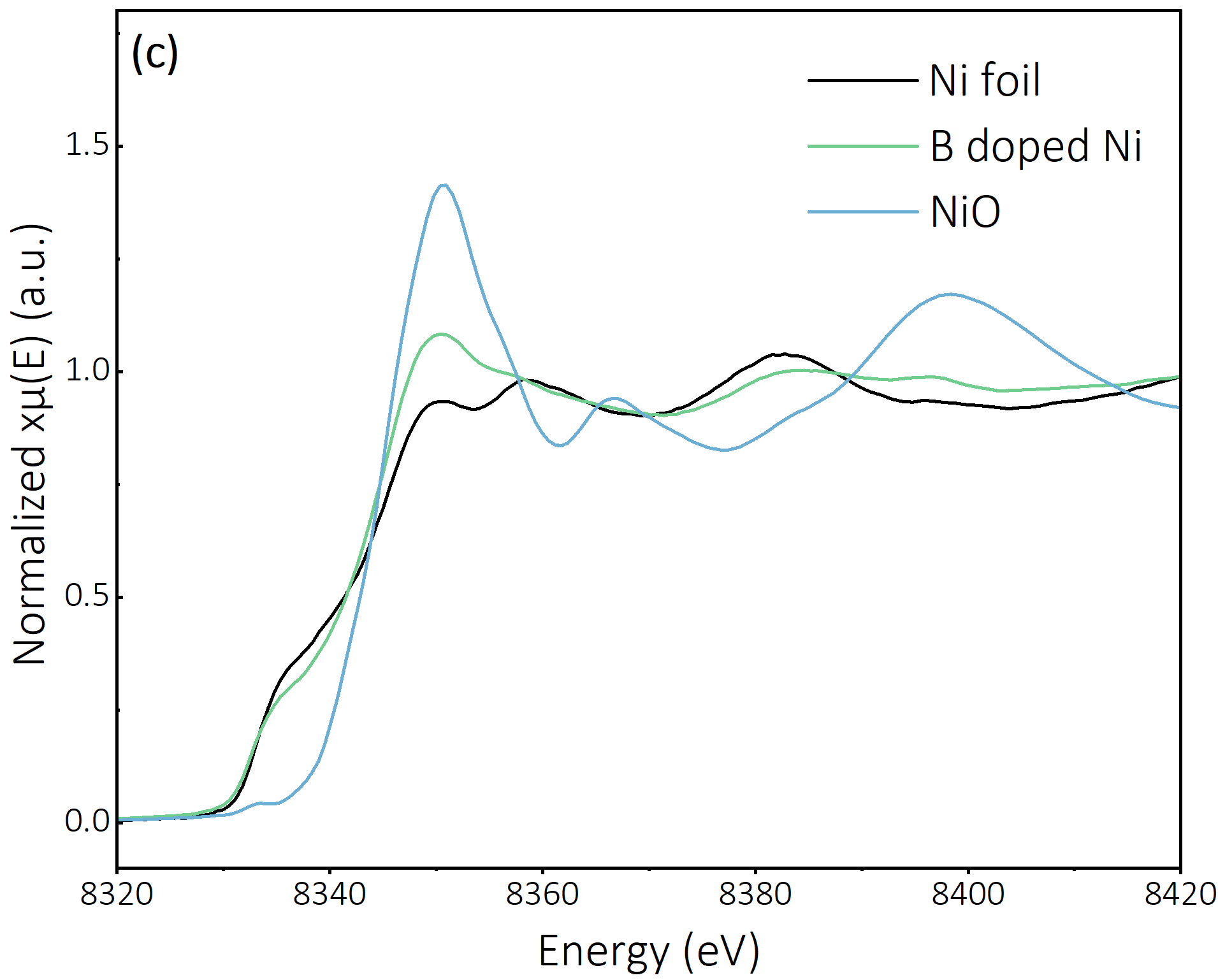

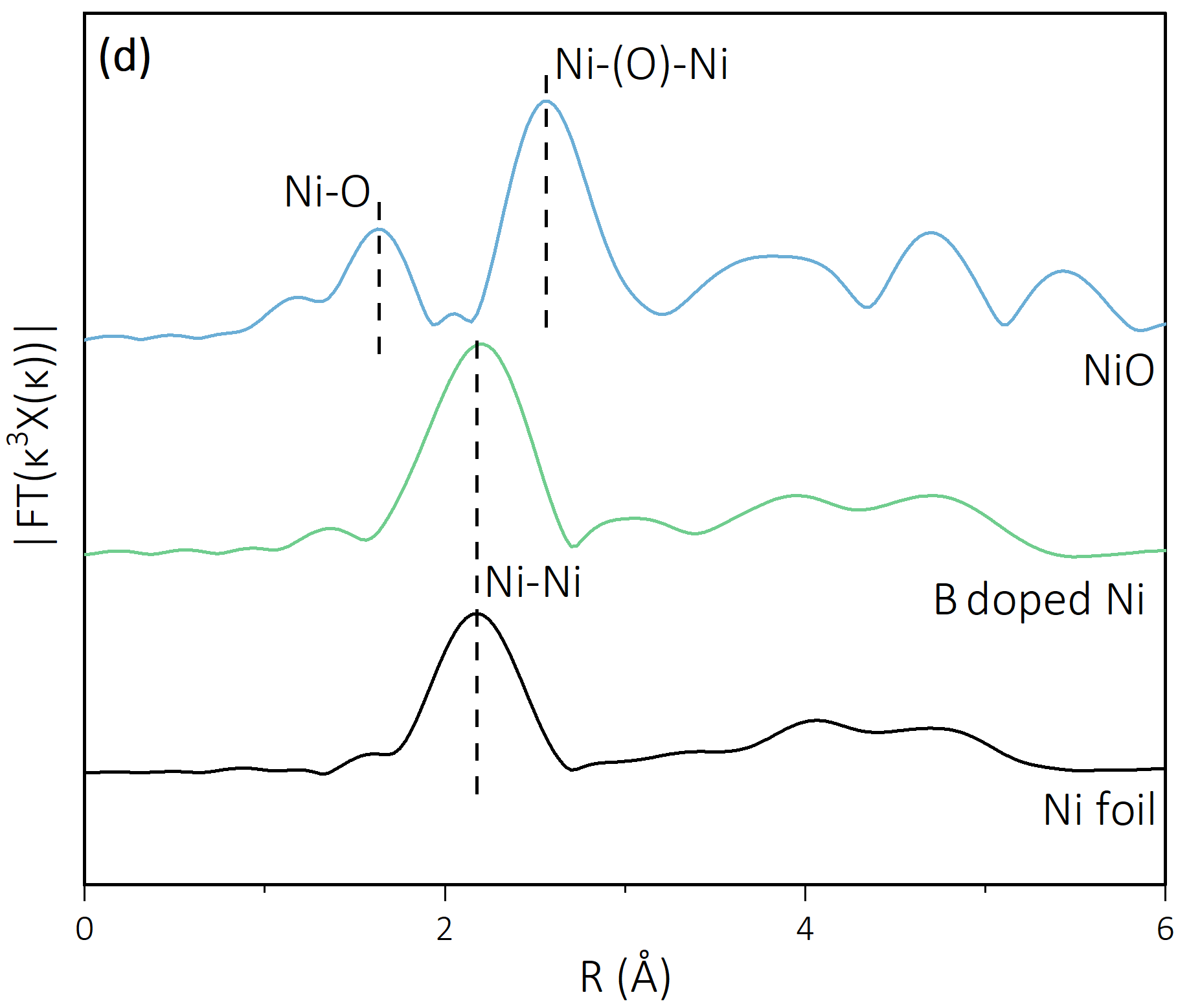


**Fig.S3 High-resolution XPS profiles of (a) Ni 2p and (b) B 1s of the Amorphous B-Ni precursor, B_2_O_3_/Ni, and B doped Ni, (c) Ni K-edge XANES and (d) EXAFS spectra of B doped Ni.**


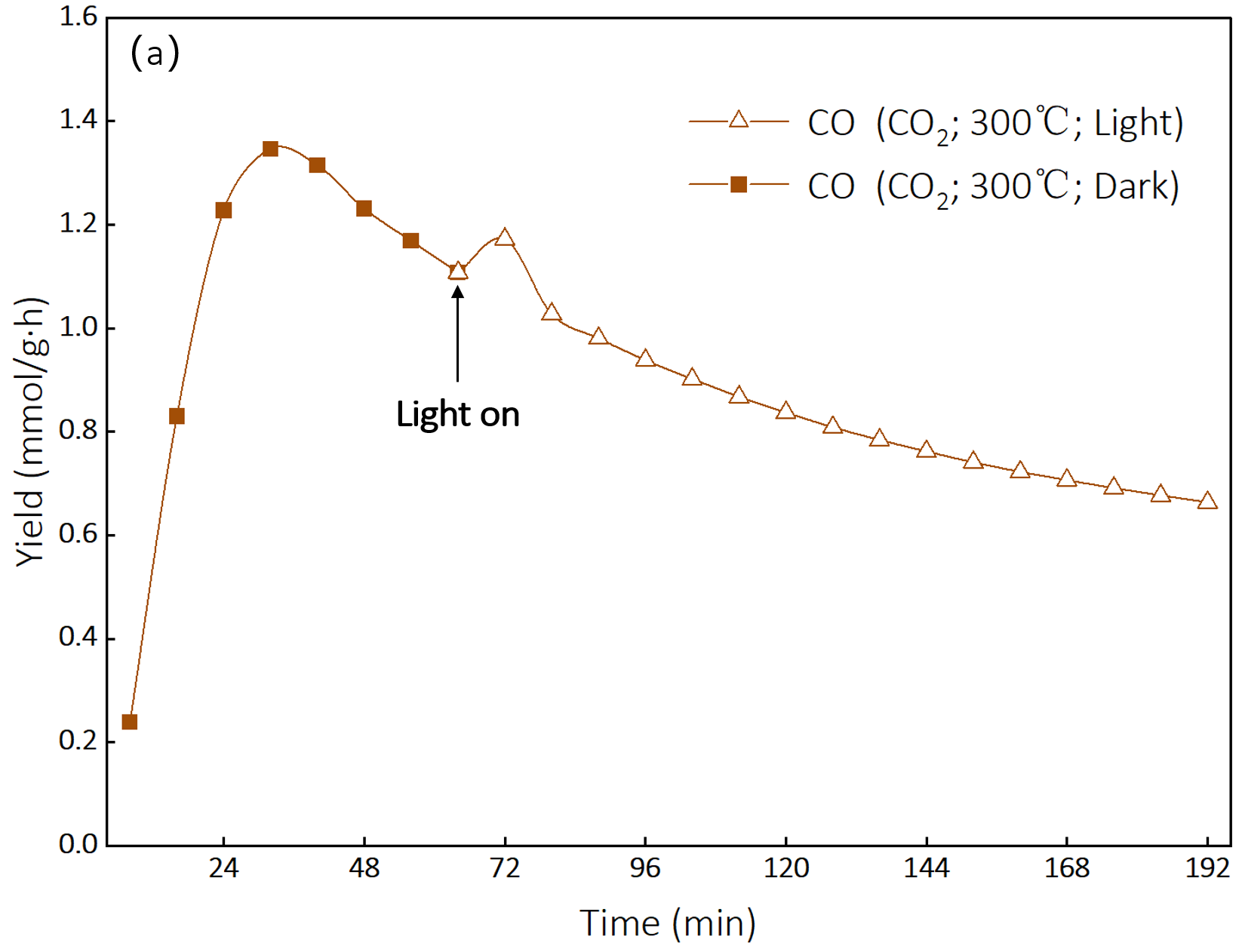


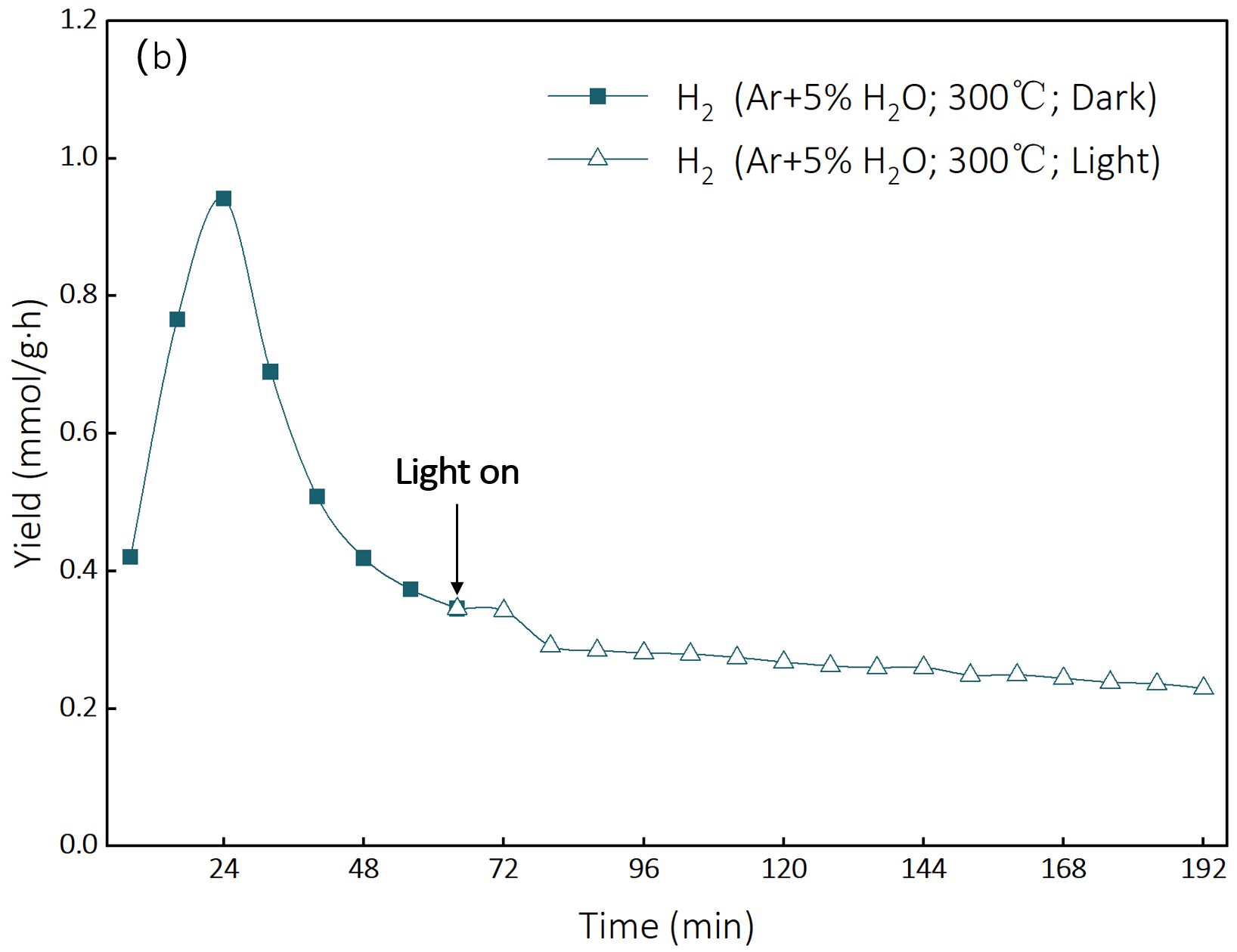


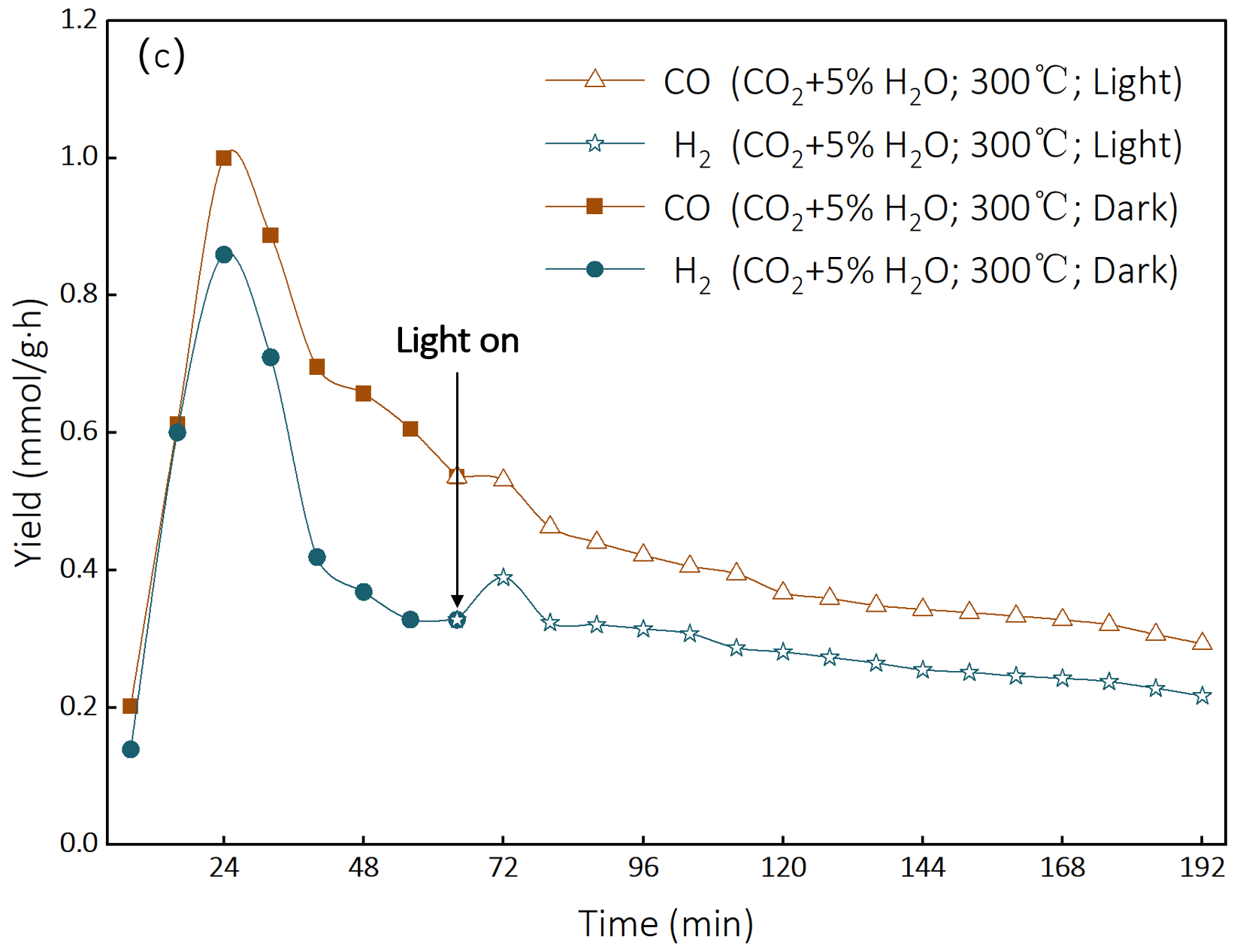


**Fig.S4 (a) CO formation from thermal dissociation and photothermal catalytic reduction of CO_2_ on B doped Ni, (b) H_2_ formation from thermal dissociation and photothermal catalytic reduction of H_2_O on B doped Ni, (c) CO and H_2_ formation from thermal dissociation and photothermal catalytic reduction of CO_2_ and H_2_O on B doped Ni.**


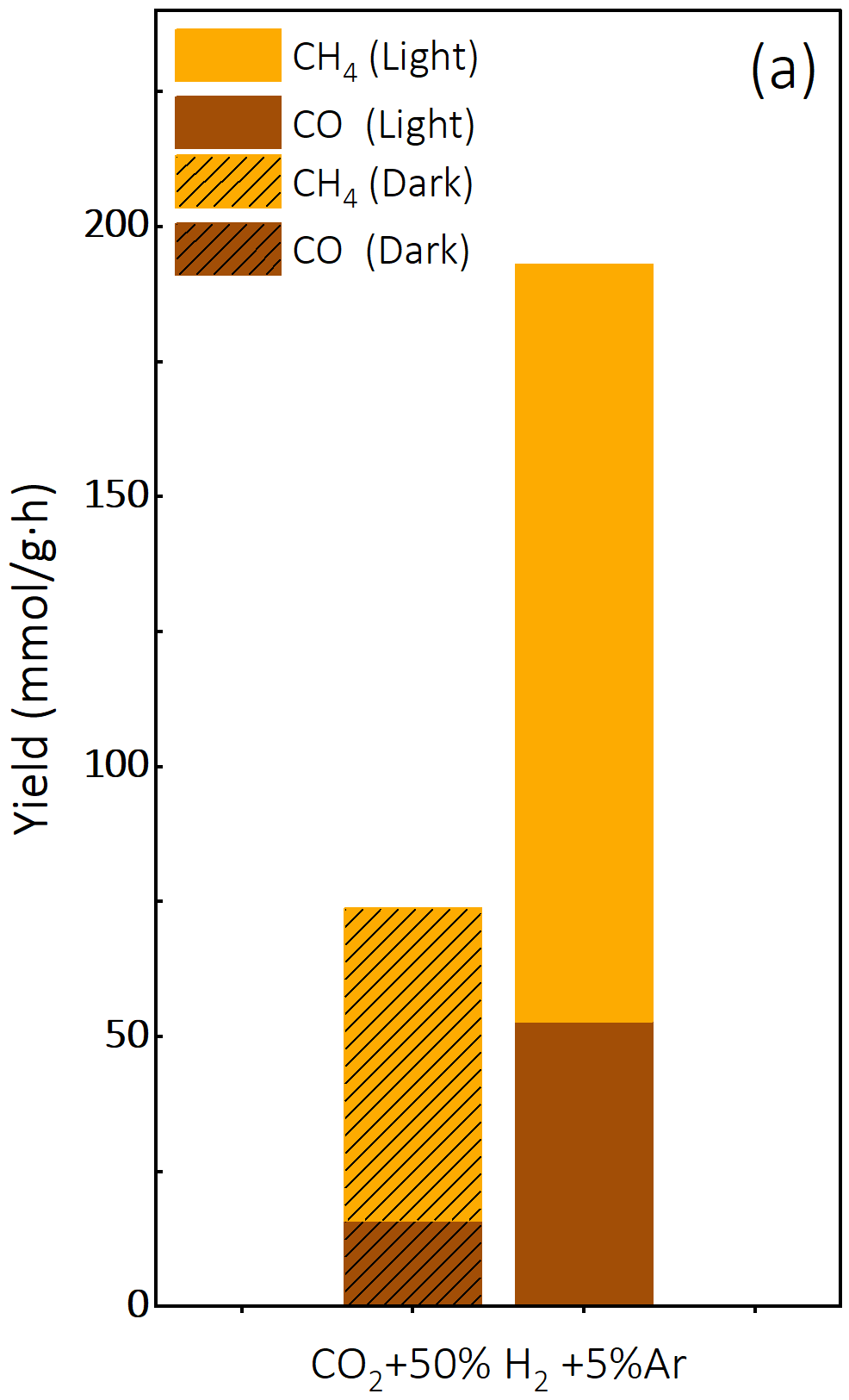

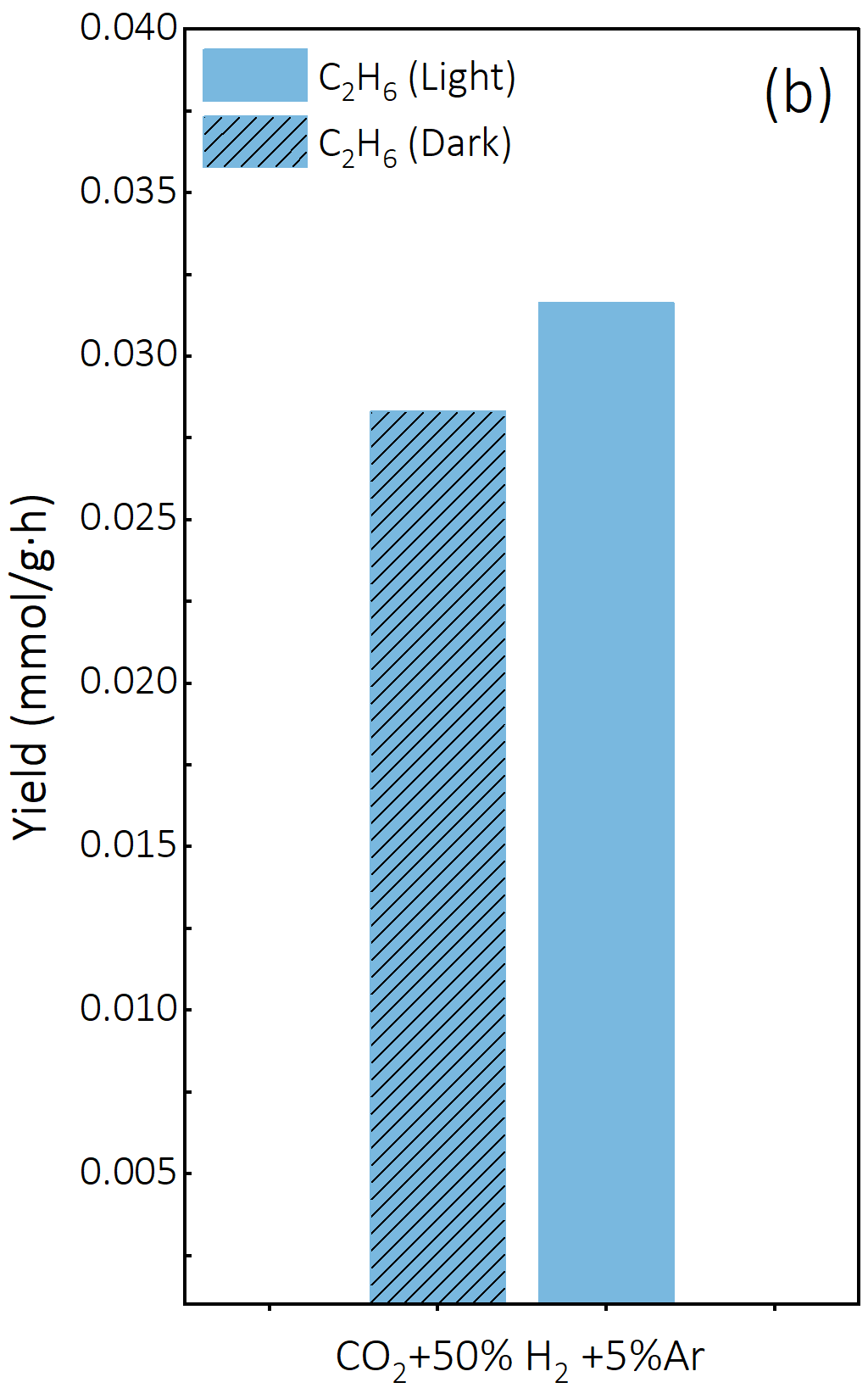


**Fig.S5 CO, CH_4_ and C_2_H_6_ yield by thermal-assisted photothermal catalytic reduction (800mW/cm2) reaction on B doped Ni with 5%Ar.**

| 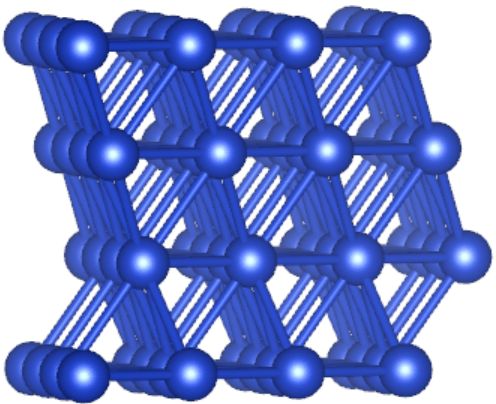  Ni (111) | 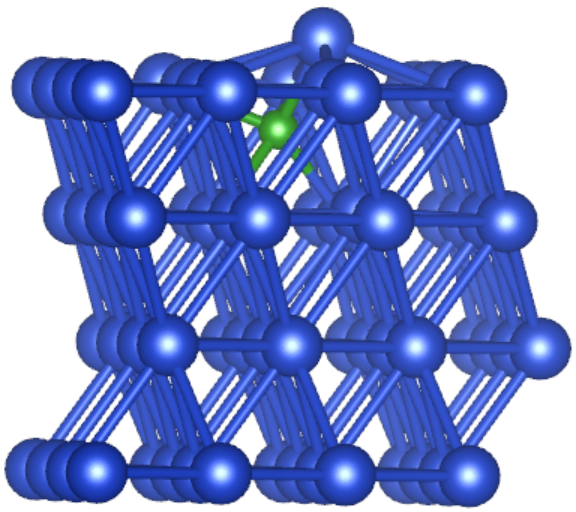  1B-doped Ni (111) | 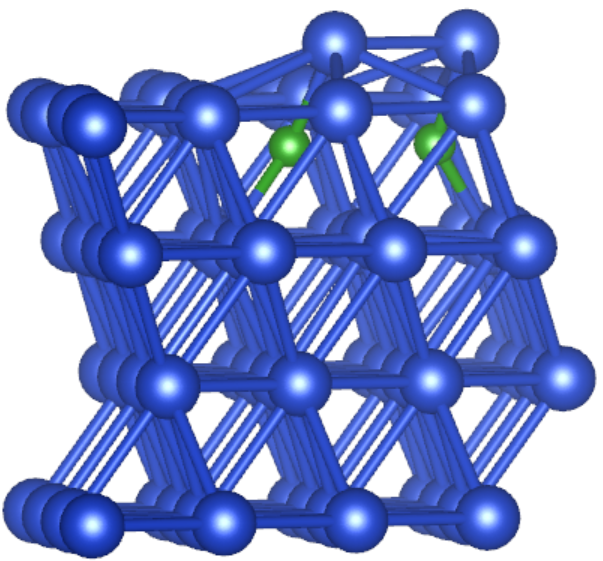  2B-doped Ni (111) |
| --- | --- | --- |
|  | 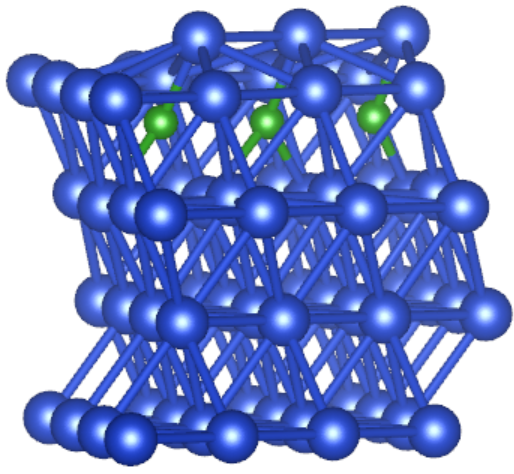  3B-doped Ni (111) | 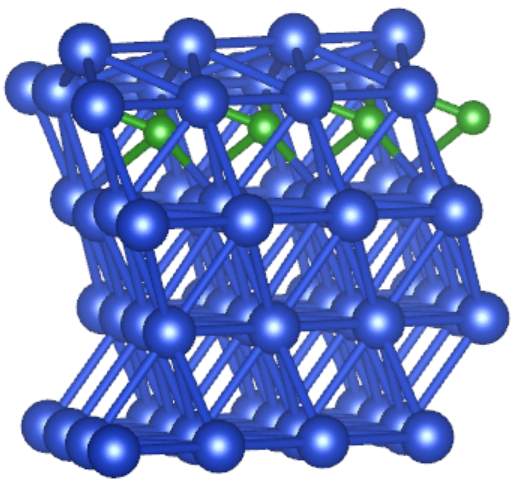  4B-doped Ni (111) |

**Fig.S6 The crystal structure of Ni (111) and B-doped Ni (111).**

**Table S1 The optimized geometries for the reaction intermediates during the CO_2_ reduction process to CH_4_ product over B doped Ni(111) surface.**

| 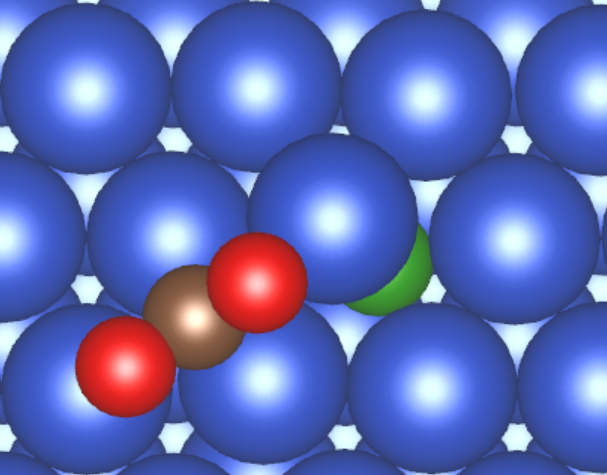  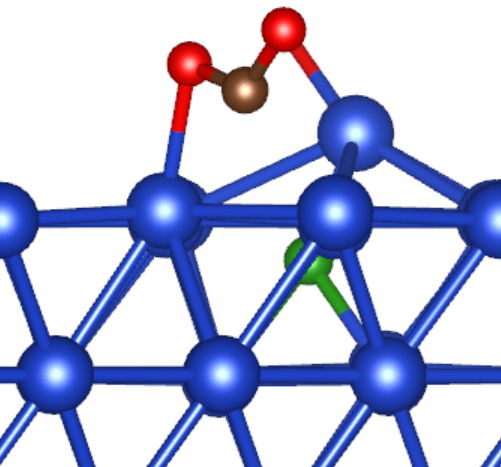  CO_2_ | 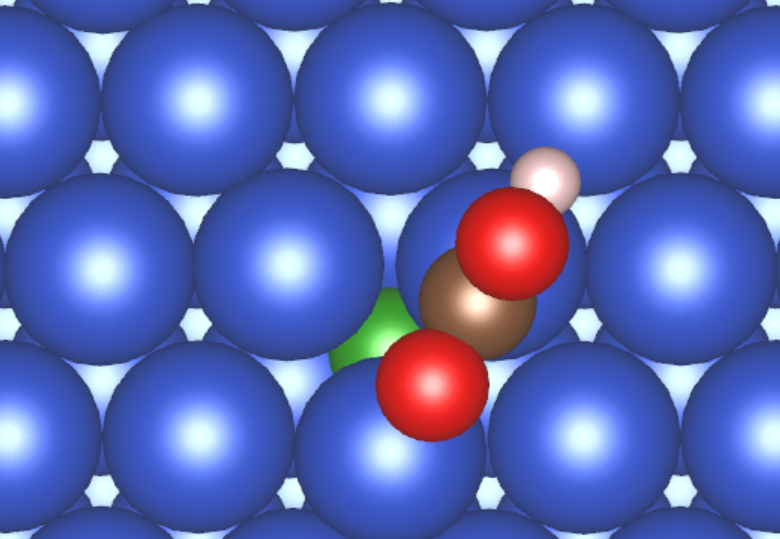  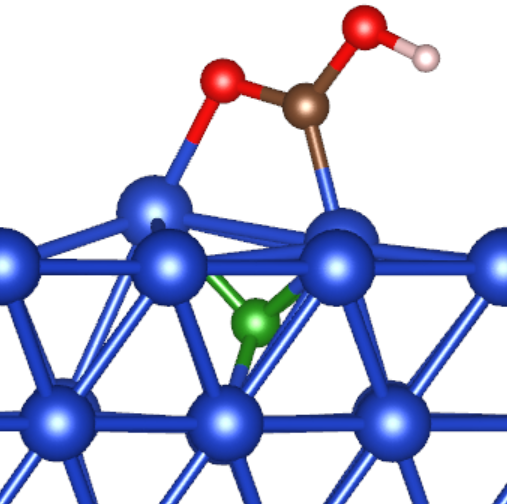  COOH | 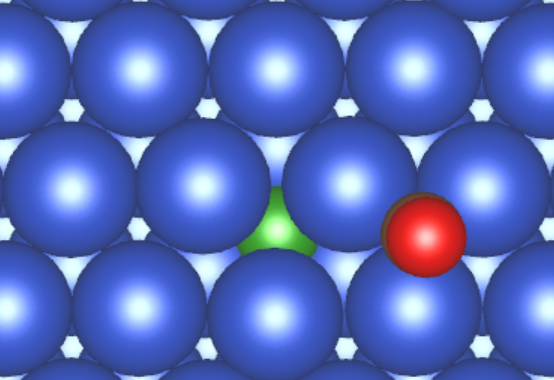  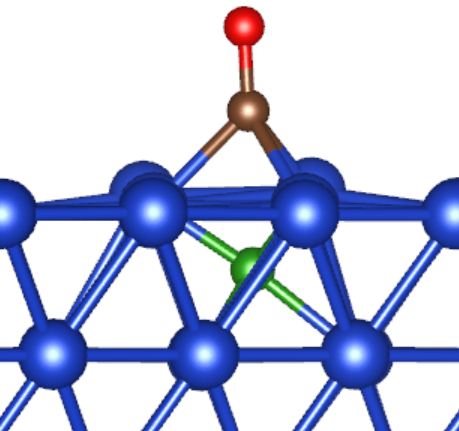  CO |
| --- | --- | --- |
| 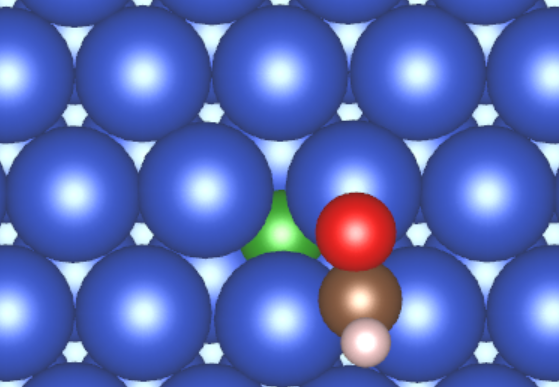  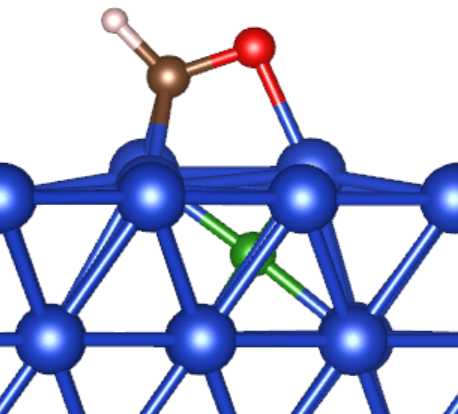  CHO | 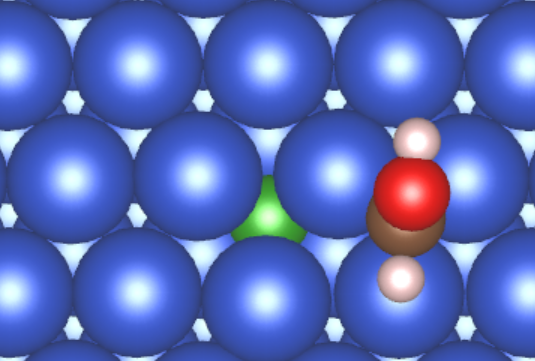  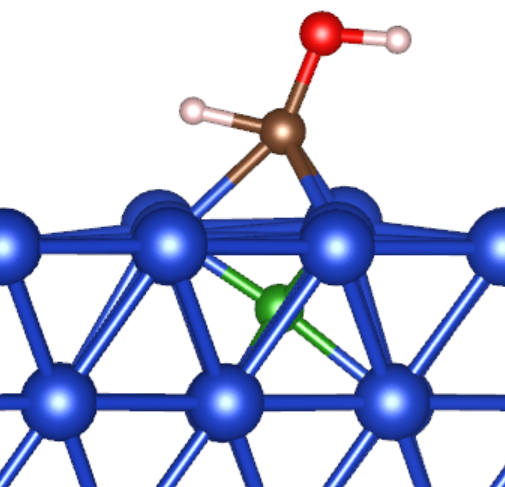  CHOH | 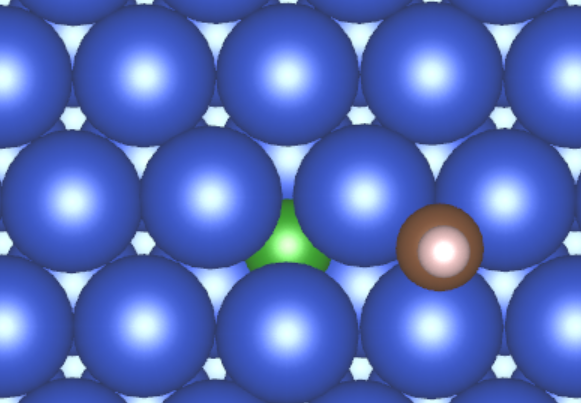  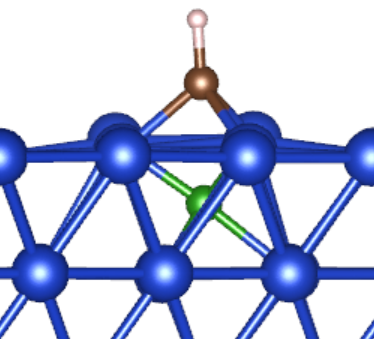  CH |
| 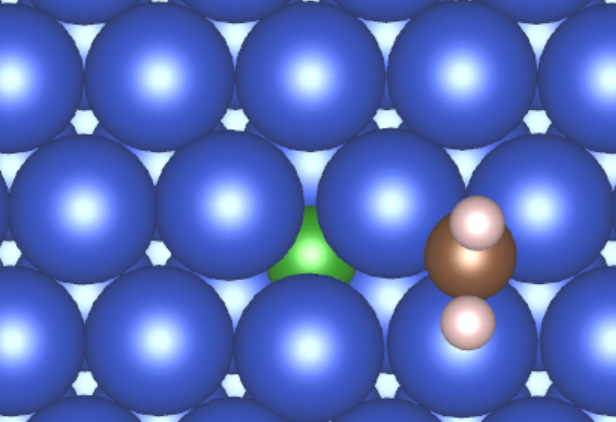  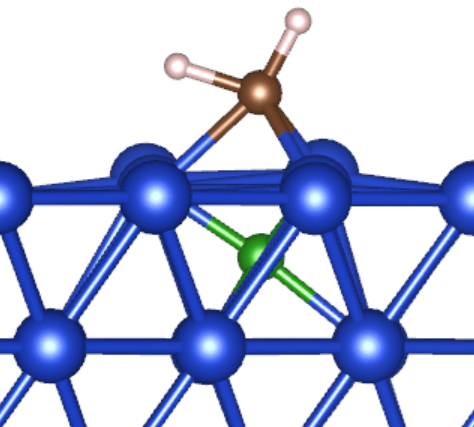  CH_2_ | 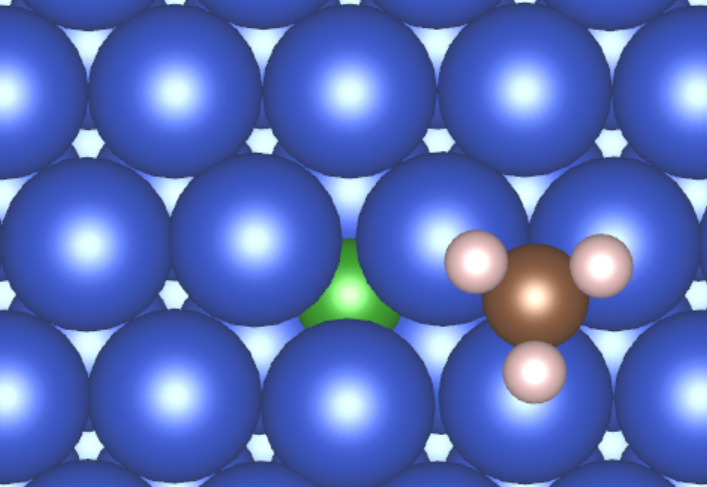  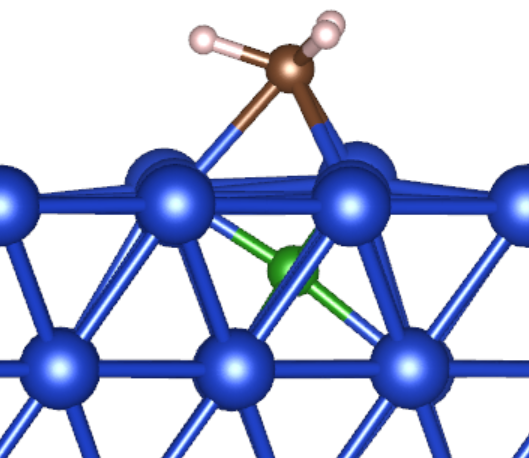  CH_3_ | 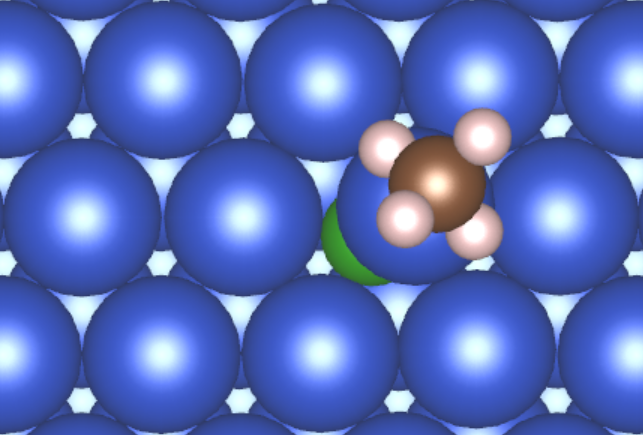  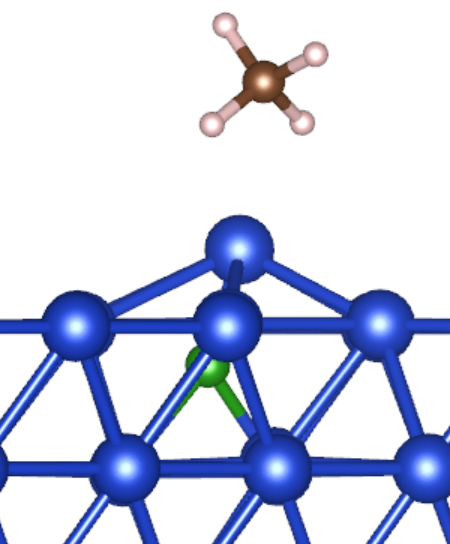  CH_4_ |
| 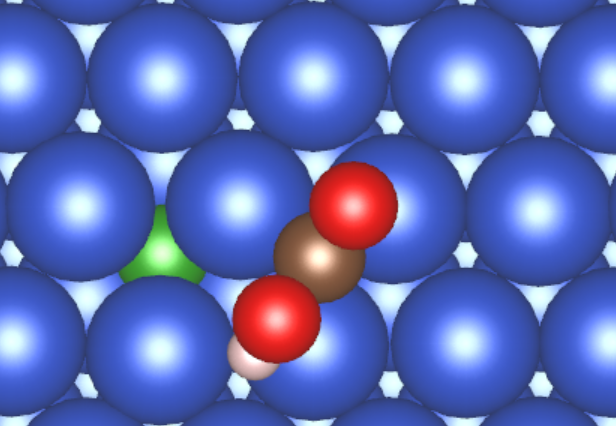  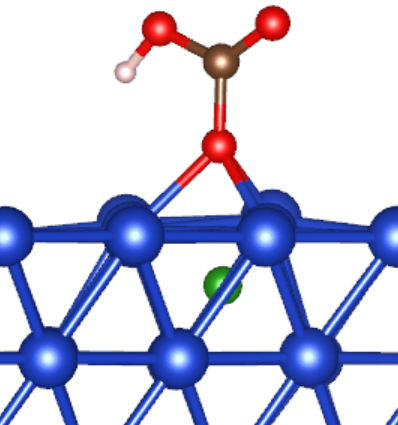  HCO_3_ | 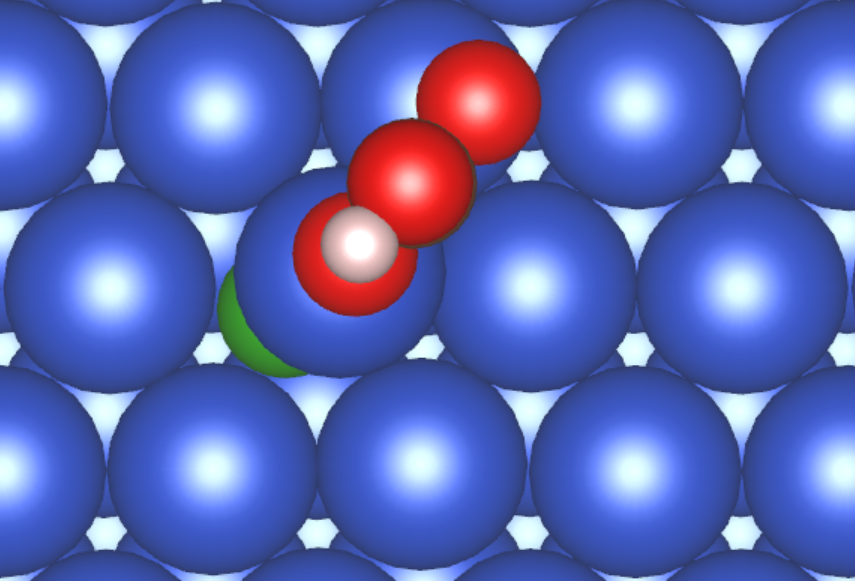  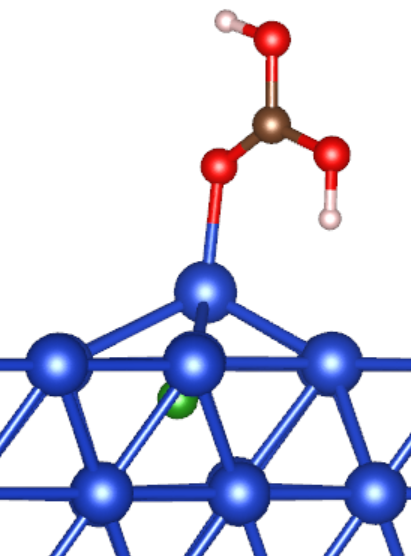  H_2_CO_3_ |  |

Different B doping site on Ni(111) surface

The B atom doped at the subsurface site on Ni(111) surface is the most favorable configuration, which is 0.45eV and 0.53eV more stable than that at the hcp and fcc sites.

| 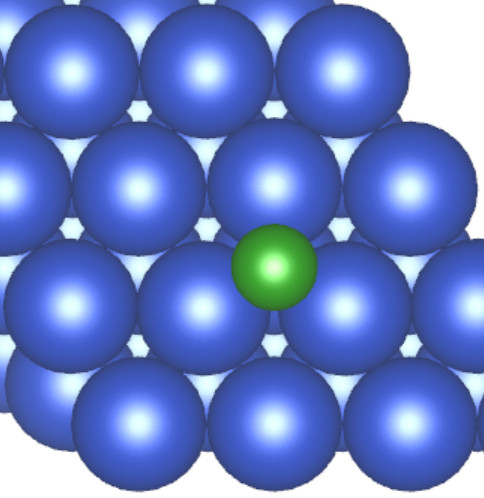 | 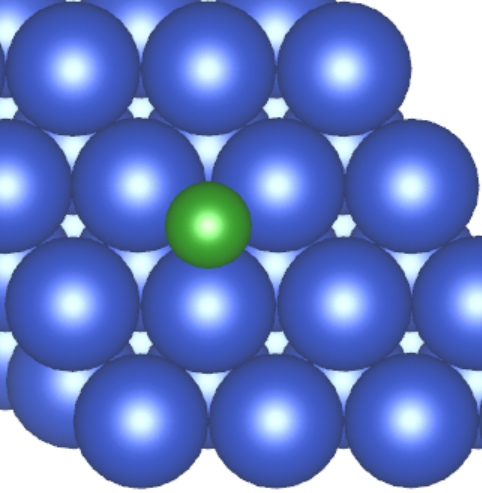 | 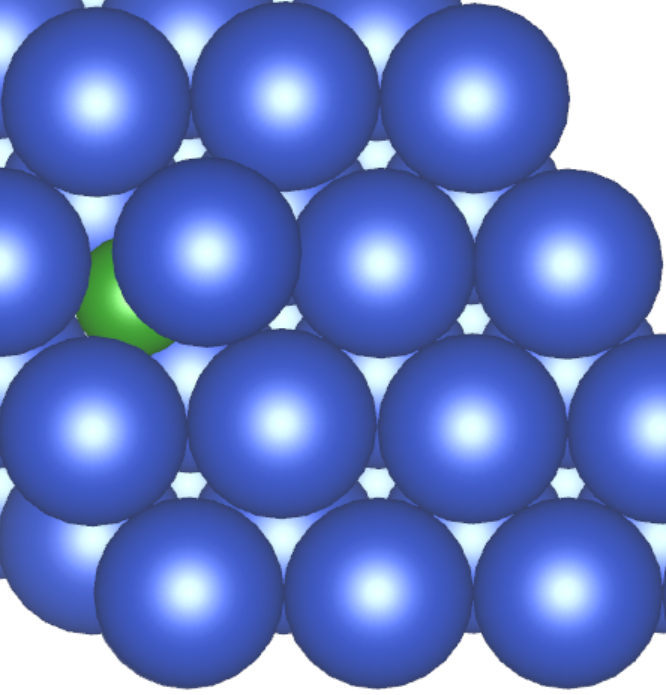 |
| --- | --- | --- |
| 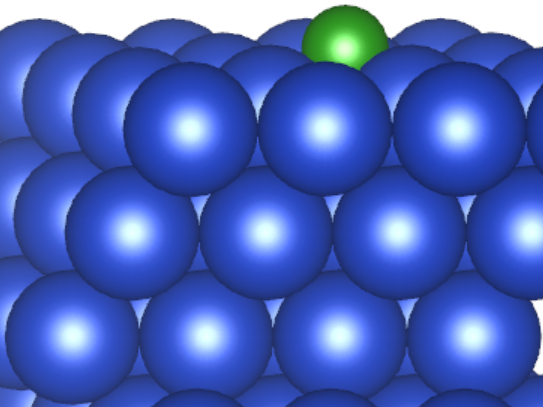 | 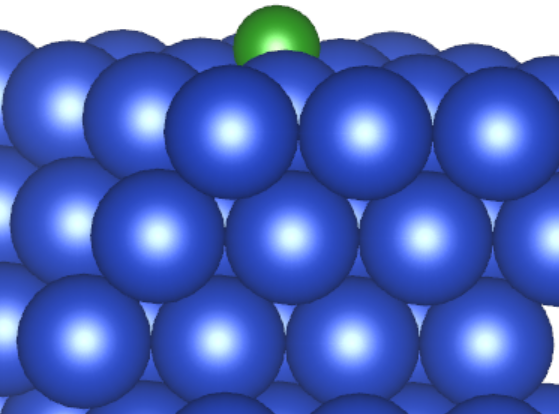 | 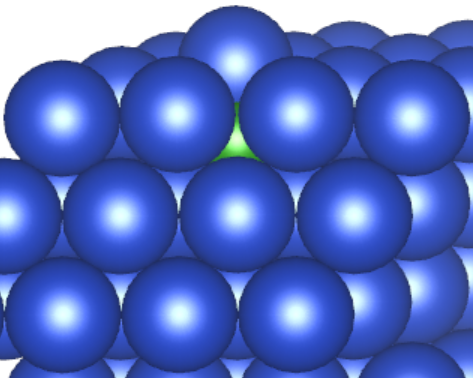 |
| hcp | fcc | subsurface |

**Fig.S7 The DFT model of Ni(111) surface with B doped at different sites.**

**Table S2 The formation energy of the CO_2_ reduction to CH_4_ reaction-involved intermediates at various adsorption sites on Ni(111) surface.**

| Species | Adsorption Sites | Formation Energy (eV) |
| --- | --- | --- |
| CO_2_ | top | 0.35 |
|  | fcc | 0.48 |
|  | hcp | 0.49 |
| COOH | bridge | -0.17 |
| HCO_3_ | fcc | 0.33 |
|  | hcp | 0.42 |
| H_2_CO_3_ | top | -0.19 |
|  | bridge | 0.16 |
| CO | top | -0.66 |
|  | fcc | -1.01 |
|  | hcp | -1.26 |
| CHO | top | -0.11 |
|  | hcp | -0.49 |
|  | fcc | -0.27 |
| CHOH | top | -0.02 |
|  | fcc | -0.11 |
|  | hcp | -0.6 |
| CH | fcc | -0.89 |
|  | hcp | -1.11 |
| CH_2_ | fcc | -1.07 |
|  | hcp | -1.27 |
| CH_3_ | fcc | -1.67 |
|  | hcp | -1.91 |
|  | top | -1.32 |
| CH_4_ | hcp | -2.46 |
|  | fcc | -2.17 |

**Table S3 The formation energy of the CO_2_ reduction to CH_4_ reaction-involved intermediates at various adsorption sites on B doped Ni(111) surface.**

| Species | Adsorption Sites | Formation Energy (eV) |
| --- | --- | --- |
| CO_2_ | fcc-nearB | 0.17 |
|  | hcp-nearB | -0.26 |
|  | top-nearB | -0.12 |
|  | top | -0.12 |
|  | hcp | 0.22 |
|  | fcc | 0.24 |
| COOH | fcc-nearB | -0.51 |
|  | hcp-nearB | -0.49 |
|  | top-nearB | -0.52 |
|  | bridge | -0.47 |
|  | fcc | -0.39 |
| HCO_3_ | fcc-nearB | 0.04 |
|  | hcp-nearB | 0.15 |
|  | hcp | 0.15 |
|  | fcc | 0.1 |
| H_2_CO_3_ | top-nearB | -0.79 |
|  | top | -0.47 |
|  | bridge | -0.14 |
| CO | top-nearB | -0.67 |
|  | fcc-nearB | -1.37 |
|  | hcp-nearB | -1.35 |
|  | fcc | -1.31 |
|  | hcp | -1.31 |
|  | top | -0.94 |
| CHO | hcp-nearB | -0.62 |
|  | top-nearB | -0.19 |
|  | fcc-nearB | -0.59 |
|  | top | -0.36 |
|  | hcp | -0.55 |
| CHOH | top-nearB | -0.34 |
|  | fcc-nearB | -0.75 |
|  | hcp-nearB | -0.65 |
|  | fcc | -0.67 |
|  | hcp | -0.53 |
|  | top | -0.33 |
| CH | hcp-nearB | -1.14 |
|  | fcc-nearB | -1.26 |
|  | fcc | -1.19 |
|  | hcp | -1.19 |
| CH_2_ | hcp-nearB | -1.33 |
|  | fcc-nearB | -1.43 |
|  | fcc | -1.34 |
|  | hcp | -1.3 |
| CH_3_ | top-nearB | -1.75 |
|  | fcc-nearB | -1.99 |
|  | hcp-nearB | -1.95 |
|  | fcc | -1.95 |
|  | hcp | -1.9 |
|  | top | -1.63 |
| CH_4_ | top-nearB | -2.52 |
|  | fcc-nearB | -2.48 |
|  | hcp-nearB | -2.48 |
|  | fcc | -2.47 |
|  | hcp | -2.48 |
|  | top | -2.47 |

**Table S4 CO_2_ adsorption energy on Ni(111) and B doped Ni(111) based on DFT calculations.**

| Surface | Adsorption site | *E_ads_* (eV) |
| --- | --- | --- |
| Ni(111) | fcc | 0.48 |
|  | hcp | 0.49 |
|  | top | 0.35 |
| B doped Ni(111) | fcc-nearB | 0.17 |
|  | hcp-nearB | -0.26 |
|  | top-nearB | -0.12 |
|  | top | -0.12 |
|  | hcp | 0.22 |
|  | fcc | 0.24 |

**Table S5 H_2_O adsorption energy on Ni(111) and B doped Ni(111) based on DFT calculations.**

| Surface | Adsorption site | *E_ads_* (eV) |
| --- | --- | --- |
| Ni(111) | top | -0.11 |
| B doped Ni(111) | top-nearB | -0.63 |
|  | top | -0.41 |

**Table S6 Dissociative adsorption energy of CO* and O* on Ni(111) and B doped Ni(111) based on DFT calculations.**

| Surface | Adsorption site  (CO*-O*) | ΔEchem (eV) |
| --- | --- | --- |
| Ni(111) | hcp-hcp | -0.73 |
|  | fcc-fcc | -0.85 |
| B doped Ni(111) | fcc-fcc | -1.12 |
|  | top-hcp | -0.7 |
|  | hcp-fcc | -1.2 |

**Table S7 Dissociative adsorption energy of OH* and H* on Ni(111) and B doped Ni(111) based on DFT calculations.**

| Surface | Adsorption site  （OH*-H*） | Δ*E_chem_* (eV) |
| --- | --- | --- |
| Ni(111) | fcc-hcp | -0.54 |
| B doped Ni(111) | fcc-fcc | -0.58 |

**Table S8 Dissociative adsorption energy of O*, H* and H* on Ni(111) and B doped Ni(111) based on DFT calculations.**

| Surface | Adsorption site  (O*-H*-H*) | Δ*E_chem_* (eV) |
| --- | --- | --- |
| Ni(111) | hcp-hcp-hcp | -0.65 |
| B doped Ni(111) | fcc-fcc-fcc | -0.92 |


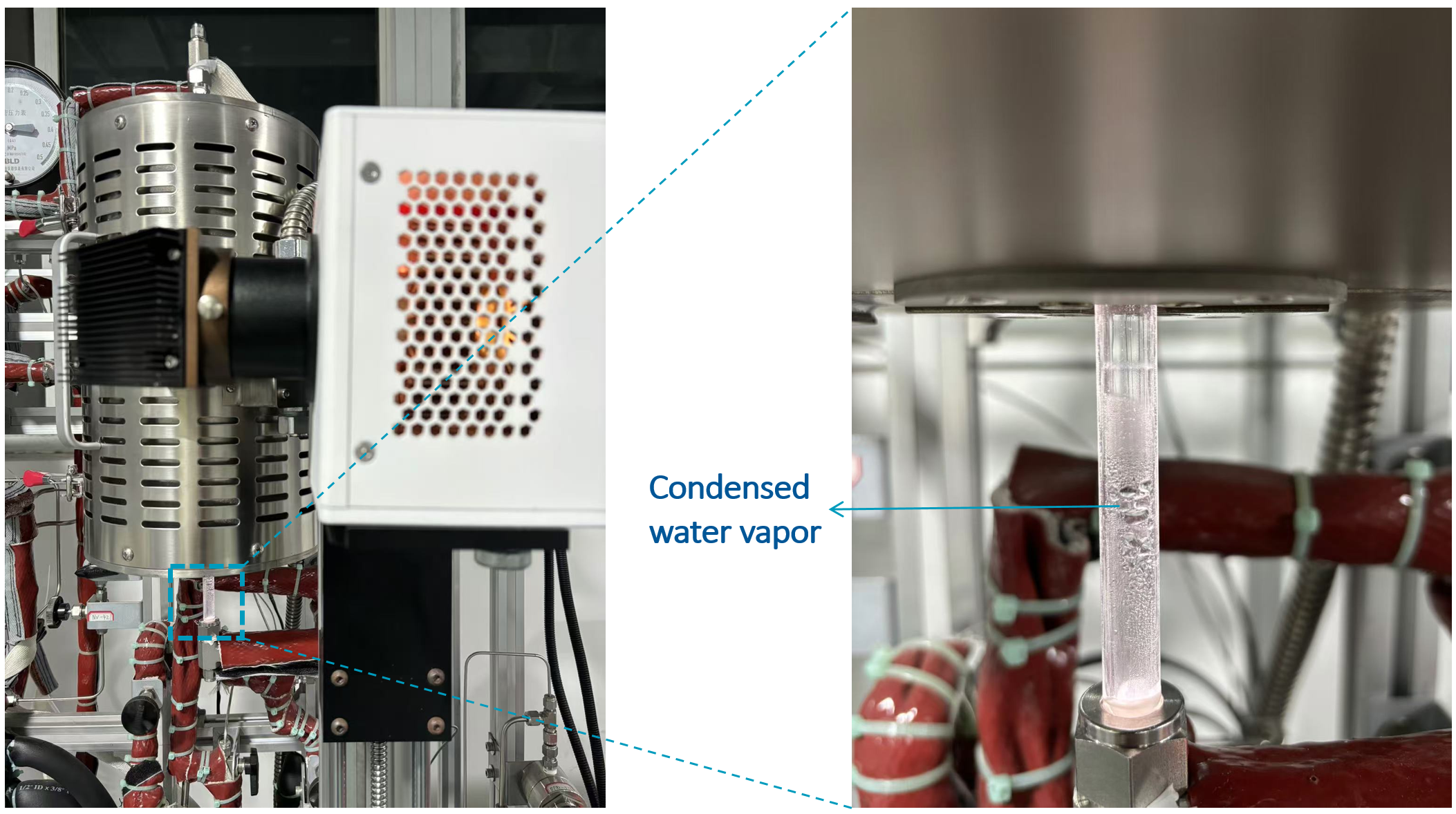


**Fig.S8 Experimental setup for CO_2_ reduction with H_2_ on B doped Ni.**
